# Antioxidants of Fruit Extracts as Antimicrobial Agents against Pathogenic Bacteria

**DOI:** 10.3390/antiox11030602

**Published:** 2022-03-21

**Authors:** Sureeporn Suriyaprom, Pascale Mosoni, Sabine Leroy, Thida Kaewkod, Mickaël Desvaux, Yingmanee Tragoolpua

**Affiliations:** 1 Department of Biology, Faculty of Science, Chiang Mai University, Chiang Mai 50200, Thailand; sureeporn_suriyaprom@cmu.ac.th (S.S.); thida_kaewkod@cmu.ac.th (T.K.); 2 Graduate School, Chiang Mai University, Chiang Mai 50200, Thailand; 3 Microbiologie Environnement Digestif et Santé (MEDiS) UMR454, INRAE, Université Clermont Auvergne, 63000 Clermont-Ferrand, France; pascale.mosoni@inrae.fr (P.M.); sabine.leroy@inrae.fr (S.L.); 4 Research Center in Bioresources for Agriculture, Industry, and Medicine, Faculty of Science, Chiang Mai University, Chiang Mai 50200, Thailand

**Keywords:** antimicrobial activity mechanisms, antioxidant properties, fruit extracts, organic acids, polyphenols, terpenes

## Abstract

Fruit is an essential part of the human diet and is of great interest because of its richness in phytochemicals. Various fruit extracts from citrus, berries and pomegranates have been shown to possess a broad spectrum of medicinal properties. Fruit phytochemicals are of considerable interest because of their antioxidant properties involving different mechanisms of action, which can act against different pathogenic bacteria. The antioxidant capacity of fruit phytochemicals involves different kinds of reactions, such as radical scavenging and chelation or complexation of metal ions. The interaction between fruit phytochemicals and bacteria has different repercussions: it disrupts the cell envelope, disturbs cell–cell communication and gene regulation, and suppresses metabolic and enzymatic activities. Consequently, fruit phytochemicals can directly inhibit bacterial growth or act indirectly by modulating the expression of virulence factors, both of which reduce microbial pathogenicity. The aim of this review was to report our current knowledge on various fruit extracts and their major bioactive compounds, and determine the effectiveness of organic acids, terpenes, polyphenols, and other types of phenolic compounds with antioxidant properties as a source of antimicrobial agents.

## 1. Introduction

Infectious diseases are the world’s biggest killer and involve the invasion and replication of microorganisms in the body, such as bacteria, viruses, fungi, or parasites [1]. Most infections are caused by bacteria, and the symptoms depend on the area of infection in the body. Among the pathogenic bacteria that contribute to significant diseases or infections, *Staphylococcus* and *Streptococcus* bacteria are responsible for skin infections [2]. *Escherichia coli*, *Listeria monocytogenes*, *Salmonella*, and *Vibrio* are highlighted as causes of foodborne illness [3]. In addition, uropathogenic *E. coli* (UPEC)*,* *Klebsiella*, *Proteus*, and *Enterococcus faecalis* are the major cause of urinary tract infections [4]. Different pathogenic microorganisms use general strategies for causing infection and disease [5]. Most bacterial infections are usually treated with antibiotics. However, in the last 3 decades, natural compounds have been studied more frequently, providing increased evidence of antibacterial activity worldwide [6]. Thousands of compounds isolated from plants were also shown to have antimicrobial or medicinal properties [7].

Fruits are naturally rich sources of phytochemicals that are used for both consumption and natural antimicrobial agents [8]. Some phytochemicals or chemicals in fruit extracts play important roles in protecting fruit from insects and microbes, as well as from stressful conditions such as ultraviolet (UV) light and drastic temperatures [9]. Fruit phytochemicals are used extensively for their medicinal value and are composites of several components, which can be classified into large groups according to their chemical structure [6], such as organic acids, terpenes, and polyphenols.

Phytochemicals in fruit extracts are recognized for their antioxidant and antibacterial activity. Various fruits have received much attention for their antibacterial efficacy because they are considered safe for human use [10]. Berry extracts were the most studied for their inhibitory effects against pathogenic bacteria. In particular, cranberries and blueberries contain condensed tannins (proanthocyanidins) that can prevent symptomatic urinary tract infections (UTIs), which are caused by uropathogenic strains of *E. coli* in nearly 95% of cases [11,12,13]. Phenolic compounds present in berries and commonly consumed in Europe are effective against *E. coli*, which is a major cause of diarrhea and is responsible for extraintestinal infections [14]. Moreover, phenolic compounds were shown to be active against intestinal pathogens such as *Salmonella* and *Campylobacter* [10]. Fruit-derived antimicrobial agents, known as secondary metabolites, play a role not only by inhibiting pathogens, but also by preventing chronic diseases. Multiple fruits such as grapes, berries, pomegranates, and apples have been reported to prevent cardiovascular disease [15], Alzheimer’s disease [16], cancer, inflammation, aging, viral infections [17], allergy and cutaneous damage including skin carcinogenesis [18]. Most of their health benefits are due to their high antioxidant activity [8]. So far, many phytochemicals with different mechanisms of action have been identified as antibacterial compounds. Several studies have shown that phytochemicals interact with and have synergetic effects against pathogenic bacteria through different mechanisms of action [6]; however, the exact mechanisms of action remain unknown [19]. This review emphasizes the main bioactive compounds found in fruit extracts and focuses on the antioxidants that have been reported to act as antimicrobial agents against pathogenic bacteria.

## 2. Components of Fruit Extracts as Antimicrobial Agents

Numerous studies have been conducted in vitro and in vivo on the efficacy of fruit phytochemicals as antimicrobial agents. This review provides an overview of the in vitro antimicrobial properties of some major group of phytochemicals, including organic acids, terpenes, and polyphenols, as well as other types of phenolic compounds (Table 1).

### 2.1. Organic Acids

Most fruits have acidic pH due to their content in organic acids. Organic acids are weak acids that dissociate in an aqueous solution that releases one or more protons. In most organic acids, acidity arises from the dissociation of a carboxylic acid group, except in the case of ascorbic acid [43]. Depending on the species and cultivars, the organic acids naturally found in fruits can be present in different quantities, which add variance to the fruits’ taste and flavor [43]. In unripe fruits, the organic acid content is higher than that of sugar, resulting in a sour flavor. These acids aid in protection from animals and microorganisms [23]. The biochemical nature of organic acids naturally found in fruits can be quite diverse, such as benzoic acid in cranberries or sorbic acid in rowanberries [8,44]. Citric acid (1–6%), naturally occurring in citrus fruits, was reported to reduce the growth of pathogenic bacteria, namely *Staphylococcus aureus, Shigella dysenteriae* and *E. coli* isolated from rejections of slaughterhouses and wastewater [20]. Citric acid significantly affects the elasticity of *Pseudomonas aeruginosa* biofilm, which has mechanical properties that are highly resistant to chemical disturbances [21].

Malic acid (2.6%), present in apples, blackberries, cherries, apricots, peaches, mangos, and plums, appears to be the strongest antimicrobial acid for prevention of growth of *L. monocytogenes, E. coli* O157:H7, and *Salmonella enterica* serovar Gaminara [22]. Berries have an exceptionally low pH (pH 2.7–3.5) and sugar content, causing an acidic flavor. Their major organic acids, citric and malic acids, are consistently reported on, whereas benzoic acid (0.1–0.7 g/L) is primarily present in lingonberries, cranberries, cloudberries, and black crowberries [45]. Propionic acid is naturally found in apples and strawberries [46]; 0.3% of sodium salt of the propionic acid was reported to act as an antimicrobial agent by inducing a growth delay in *S. aureus, Sarcina lutea, L. monocytogenes* and *Proteus vulgaris* [23]. Compared with citric, malic, and lactic acid found in grapes, tartaric acid (2.6%) appears to be the most efficient organic acid against *L. monocytogenes, S.* Gaminara, and *E. coli* O157:H7 [22]. Moreover, tartaric acid, citric acid and acetic acid observed in various wild berry fruits such as cranberries, bilberries, blueberries, blackberries, raspberries, black chokeberries, red currants, and black currants were shown to inhibit the growth of *S. enterica* serovar Typhimurium at concentrations of 0.312%, 0.625%, and 1.250% for the three levels of strain at 10, 100, and 1000 CFU/mL, respectively [24]. Moreover, salicylic acid in berry fruits was demonstrated to inhibit *S. aureus, E. coli, P. aeruginosa* and *E. faecalis* at a minimum inhibitory concentration (MIC) between 250 and 500 µg/mL [25]. In addition, the dominant organic acid content in cranberries is found to be citric acid. It accounts for 37–64% of the total acids, while malic acids and quinic acids account for 8–43% and 11–32%, respectively [47]. The report by El Baaboua et al. [24] revealed that an increase in pH could reduce the inhibition of *S.* Typhimurium. Before neutralization, the inhibitory concentration of citric acid against *S.* Typhimurium was 0.312% (*v*/*v*), and the dosage was increased to 0.625% (*v*/*v*) after the addition of neutralizer. Puupponen-Pimiä et al. [10] showed that the critical pH values for the growth of *Staphylococcus* and *Salmonella* bacteria were 5–5.5. After adding berry extracts containing organic acids in culture media, the pH was decreased to 5 or lower, and resulted in lower counts of these bacteria, suggesting that the low pH in berries contributed to the antimicrobial activity.

### 2.2. Terpenes

Terpenes, or isoprenes are major factors for coloration and aroma, especially the flavor and bitterness in fruits [48]. Basically, these compounds are synthesized from isopentenyl diphosphate and dimethylallyl diphosphate. Terpenes are the primary constituents of the essential oils from citrus fruits including oranges, limes, lemons, and grapefruits [49]. Terpenes and their derivatives have several different chemical functionalities including antimicrobial activity against pathogens in some cases [26,50]. Moreover, eugenol is a terpene contributing to the aroma of fruits such as strawberries, blackberries, bananas, and citrus. Eugenol at a concentration of 0.07 mg/mL exhibited a bactericidal effect against *S.* Typhimurium within 2 h of treatment. In addition, terpineol present in apples, blueberries, and limes at a concentration of 0.12 mg/mL exhibited a bactericidal effects on the growth of *S. aureus*. Terpene derivatives containing hydrocarbons in citrus fruits such as carveol, citronellol and geraniol at a concentration of 0.25 mg/mL rapidly killed *E. coli* within 2 h of treatment [26]. Monoterpenes, thymol, (+) menthol, and linalyl acetate, which are mostly present in citrus fruits (e.g., bergamot), revealed bacteriostatic effects on both Gram-positive and Gram-negative bacteria, e.g., *S. aureus* and *E. coli.* The MICs of thymol, (+) menthol and linalyl acetate were demonstrated at 0.31, 0.62, and 1.25 mg/mL, respectively. These concentrations are considerably more toxic against *S. aureus* than *E. coli*, whereas (+) menthol is the most toxic compound against *E. coli* at an MIC of 2.5 mg/mL followed by thymol and linalyl acetate at 5 mg/mL [27]. Nonetheless, monoterpene (e.g., limonene, citronellal) and volatile sesquiterpene, which are major components of citrus essential oils [51], are generally more effective in inhibiting the growth of Gram-positive bacteria (e.g., *S. aureus, Bacillus cereus, L. monocytogenes*) than of Gram-negative bacteria (e.g., *Salmonella* sp., *E. coli* O157:H7), due to disruption of their cell permeability [52,53,54].

### 2.3. Polyphenols

Polyphenols are the largest category of phytochemicals in fruits. These compounds have one or more aromatic rings with one or more hydroxyl groups that are responsible for their antioxidant properties [9]. Fruit polyphenols are partially responsible for the overall organoleptic properties of fruits, such as flavor and color [55]. Regarding their chemical structure, polyphenols can be classified as flavonoids and non-flavonoid polyphenols (Table 2).

#### 2.3.1. Flavonoids

Flavonoids have the basic structure of a diphenylpropane (C6–C3–C6) skeleton, a carbon structure consisting of two phenyl rings (A and B) and a heterocyclic ring (C) [28]. Although many berries have abundant flavonoids, flavonoids can be found in the Rutaceae (e.g., bael and citrus fruits), the peels of which contain high amounts of flavonoids that were found to be 59.9–83.3 mg catechin equivalent/g extract [71], and in Passifloraceae families (e.g., passion fruits), with 70.1 mg quercetin equivalent/100 g [72]. Flavonoids are quite diverse and are classified into flavones, flavonols, flavanol-3-ols, isoflavones, flavanones and anthocyanins according to their chemical structure (Table 2). While flavones are mostly reported in herbs such as parsley or celery, they are also found in some citrus varieties such as oranges, grapefruits, and lemons. The bacteriostatic effect of flavones was found to inhibit the growth of *E. faecalis, E. coli,* and *P. aeruginosa* at an MIC of 500 µg/mL [25].

Flavonols are the predominant group of flavonoids and are present in berries, especially cranberries, black currants, lingonberries, blueberries and black grapes, as well as in apricots and apples, which contain more flavonols than most other fruits and vegetables [57]. In blueberries, flavonols clearly demonstrated a concentration in the peel [73]. Concentrations of flavonols vary among berries, from less than 20 mg/g extract to a concentration as high as 92 mg/g extract in sea buckthorn berries [10]. Flavonols are active not only against Gram-positive pathogens, as shown for *S. aureus*, methicillin-resistant *S. aureus* (MRSA), *S. epidermidis, S. haemolyticus,* and *S. pyogenes*, but also against Gram-negative species such as *E. coli, S.* Typhimurium and *K. pneumoniae* [28].

Flavan-3-ols are commonly found in apples, grapes, blackberries, and cranberries [9]. While 3′-O-methyldiplacol has an effect on the growth of Gram-positive bacteria including *B. subtilis, E. faecalis, L. monocytogenes, S. aureus, and S. epidermidis* at MICs of 2–4 μg/mL [29], quercetin 3-O-methyl ether (3.9 µg/mL) demonstrated effectiveness against *Helicobacter pylori* [28]. The oligomeric and polymeric flavan-3-ols are proanthocyanidins, known as condensed tannins. Proanthocyanidins are found in grapes, apples, bilberries, cranberries, strawberries, and blueberries [58]. In cranberries, they are responsible for an anti-adhesion property by acting on the colonic bacterial receptors, which prevent bacteria from binding to uroepithelium and proliferating [63,64]. Furthermore, proanthocyanidins from persimmons (*Astringent persimmon*) are used as a food additive, called Pancil^®^ PS-M. They have concentration-dependent antibacterial properties against oral polymicrobial biofilms [74]. The consumption of cranberries, which is a fruit rich in procyanidins (type A and B), is shown to reduce urinary tract infections, such as UPEC and *Candida albicans* infections, by reducing the adhesion of bacteria and the formation of biofilms [75,76,77,78]. One of the advanced mechanisms of action in the reduction of UPEC infections would be the binding of type A procyanidins to the pili of these pathogens, thus inhibiting their adhesion to epithelial cells in the bladder [79].

Isoflavones are mostly present in soy products, legumes, and some fruits such as currants and raisins [59]. At only 100 µM, genistein, a soy isoflavone, reduced the growth of *B. anthracis*, *S. aureus* and MRSA [60,61]. Flavanones are normally found in prunes and citrus fruits, e.g., lemons, oranges, and grapefruits [9]. Flavanones are effective against Gram-positive bacteria including *B. cereus, B. subtilis, E. faecalis, L. monocytogenes*, and *S. aureus* at MICs of 2–4 μg/mL [29]. More specifically, sophoraflavanone G showed significant antibiofilm activity *against S. epidermidis, S. aureus*, and *B. subtilis* at MICs of 3.1–12.5 μg/mL [28].

Anthocyanins are responsible for the colors of fruits, resulting in shades of red, blue, purple, and violet. They are mostly present in the Vitaceae (grapes) and Rosaceae (cherries, plums, raspberries, strawberries, blackberries, apples, peaches), Saxifragaceae (red and black currants), and Ericaceae families (blueberries and cranberries) [62]. Anthocyanins are particularly abundant in the peel of berries. Blueberries and bilberries contain the most complex composition of anthocyanins [80]. In fresh berries, the concentration of anthocyanins varies from 7.5 mg/100 g in red currants (*Ribes rubum*) up to 460 mg/100 g in chokeberries (*Aronia melanocarpa*) [81], with notable concentrations in bilberries and black currants (336 and 127 mg/g extract, respectively) [10]. Anthocyanin-rich extracts demonstrated effectiveness against *E. coli* and *Salmonella* at MICs of 10–400 mg/mL [30], and they can completely inhibit staphylococcal growth. The extracts are also active against both methicillin-susceptible *S. aureus* (MSSA) and MRSA at a concentration of 500 µg/mL [31]. Blueberry cultivars and bilberry fruits also display bacteriostatic antimicrobial properties against *Citrobacter freundii* and *E. faecalis* [80]. The authors suggested that the richness of anthocyanins in blueberry extract (500 µg/mL) hindered the biofilm formation of *S. aureus* and *E. coli* and prevented further adhesion of bacterial species, i.e., *P. aeruginosa, P. mirabilis* and *Acinetobacter baumannii* [31].

#### 2.3.2. Non-Flavonoid Polyphenols

Non-flavonoid polyphenols include various phenolic compounds, namely phenolic acids, stilbenes, lignans and xanthones (Table 2).

##### Phenolic Acids

Phenolic acids are mainly present in chokeberries, blueberries, cherries, and dark plums [6]. The two major subclasses of phenolic acids are hydroxybenzoic acids (C6-C1) and hydroxycinnamic acids (C6-C3). Ellagic acid, a derivative of hydroxybenzoic acids, is the major phenolic acid in berries of the *Rubus* (e.g., red raspberries, arctic brambles, and cloudberries), and *Fragaria* genera (e.g., strawberries) [10]. In blueberries, hydroxycinnamic acids are the most predominant phenolic acids, representing up to 75% of total phenolic acids present [66,67]. In cloudberries and bilberries, the content of hydroxycinnamic acids reaches 17–18 mg/g extract. The antibacterial activity of phenolic compounds has been previously reported from dry blueberry infusions containing 66–78 µg/mL of phenolic compounds, which were able to inhibit the growth of *P. aeruginosa* and *S. aureus* [82]. Concentrations of phenolic acids as low as 16 µg/mL demonstrated effectiveness against the biofilm formation of both MSSA and MRSA [31]. In blueberries and muscadines, phenolic acids (at 24 µg/mL and 46 µg/mL, respectively) could reduce the growth of *S.* Enteritidis [32]. Testing phenolic extracts from different berries, including bilberries, cloudberries, strawberries, raspberries, black currants, lingonberries and buckthorn berries, showed that only cranberries at 10 mg/mL could inhibit the growth of *L. monocytogenes* [10]. Among blueberries, acai berries, raspberries and strawberries, cranberry juice (86.6 mg gallic acid equivalent/mL) is the most potent antimicrobial agent against *S. aureus* [83]. Furthermore, phenolic-rich cranberry extract (0.1–1.0 mg/mL) exhibits antibiofilm activity with a significant effect on bacterial adhesion in the early stages of biofilm development, as found in *S. oralis*, *Actinomyces naeslundii*, *Veillonella parvula*, *Fusobacterium nucleatum*, *Porphyromonas gingivalis* and *Aggregatibacter actinomycetemcomitans* [33]. Additionally, phenolic acids from cloudberries and raspberries appeared to be the most effective inhibitors of growth against *Staphylococcus* and *Salmonella* [10]. In particular, 4-hydroxybenzoic acid and trans 4-hydroxycinnamic acid appeared in a dose-responsive curve to inhibit 50% of bacterial growth (IC_50_) in some of both Gram-positive (e.g., *S. aureus, S. epidermidis, B. subtilis*) and Gram-negative bacteria (e.g., *E. coli, S*. Typhimurium, *P. aeruginosa, P. syringae*) at IC_50_ concentrations of 100–170 and 160 µg/mL, respectively [34].

Hydrolyzable tannins are polymers of hydroxybenzoic acids and are further divided into gallotannins and ellagitannins [84]. Berries, pomegranates, grapes, persimmons, pears, and apples are rich sources of hydrolyzable tannins [65]. The antibacterial function of tannic acids is reported to be effective against *S.* Typhimurium, *S.* Enteritidis, *E. coli* and *S. aureus,* including antibiotic-resistant strains of the two last species, with a minimum inhibitory concentration ranging from 0.3 to 3 mg/mL [35,36,37]. Ellagitannins from berry extracts (at 2 mg/mL), bilberries, lingonberries, cranberries, red raspberries, cloudberries, strawberries, black currants and sea buckthorn berries exhibit powerful growth-inhibitory effects against *Staphylococcus* [10]. The antibacterial activity of tannic acid was revealed on the cell membrane by the formation of complexes that led to morphologic changes and an increase in membrane permeability [85].

##### Stilbenes

Stilbenes are commonly found in the skins of grapes, pomegranates, bilberries, blueberries and mulberries [68]. It was shown that triacid derivative 135 C had antibacterial capacity against several Gram-positive bacteria including *S. epidermidis, S. pneumoniae, S. pyogenes*, and *Micrococcus* spp., as well as MRSA [38]. Actually, compound 135 C exhibited the highest antimicrobial activity against MRSA with an MIC of 0.12–0.5 μg/mL, but was slightly less active against other tested Gram-positive bacteria.

##### Lignans

Lingonberries, strawberries, apples, cranberries, prunes, and pears are rich sources of lignans [69]. Flax seed is one of the main sources of lignans, and the corresponding extracts exhibited antimicrobial activity against *E. coli, P. aeruginosa, S. aureus*, and *B. subtilis* with MIC values ranging from 224 to 488 µg/mL [86]. Hydroxymatairesinol (HMR) lignan, a γ-butyrolactone derivative, at concentrations of 25–100 mg/mL was confirmed to be active against *S. epidermidis*, *Proteus* sp., and *Klebsiella* sp. [39].

##### Xanthones

Xanthones are mostly found in the pericarp, whole fruit, heartwood, and leaf of mangosteen (*Garcinia mangostana* Linn.) [70]. Xanthones are obtained from a semi-synthetic modification of α-mangostin at 30–100 µg/mL and possess several medicinal properties, such as antimicrobial activity against bacteria, specifically Gram-positive bacteria including *B. subtilis* and *S. aureus* and Gram-negative bacteria such as *E. coli* and *P. aeruginosa* [40,41]. Xanthones also exhibit antioxidant activity, neuro-protective activity and lipase inhibition, as well as anti-inflammatory and anti-cancer properties [41]. Dharmaratne et al. [42] indicated that antibacterial activity of *γ*-mangostin against MSSA, MRSA, vancomycin-sensitive enterococci (VSE) and vancomycin-resistant enterococci (VRE) was demonstrated at MICs of 3.13–6.25 µg/mL.

## 3. Mechanisms of Action of Fruit Extracts as Antimicrobials

The higher antimicrobial activity in fruit relates to the presence of hydroxyl groups within polyphenols, phenolic and alcohol compounds [26]. While the mechanisms of action for these phytochemical compounds remain unclear, they could interact with microbial cells at the bacterial cell envelope with consequences for transport and bacterial adhesion, as well as for enzyme activities or enterotoxin production (Table 3) [56].

### 3.1. Interaction with the Bacterial Cell Envelope

Susceptibility of bacteria to phytochemicals fluctuates with differences in the structure of the bacterial cell envelope and varies between bacteria. In a simplistic manner, bacterial cells are generally used to differentiate between Gram-positive and Gram-negative bacteria. Gram-positive bacteria display a biological membrane and a thick cell wall made of peptidoglycan, whereas the Gram-negative bacteria exhibit two biological membranes, i.e., an inner and outer membrane, with an in-between periplasmic space containing a thin peptidoglycan cell wall. For the record, the bacterial world is more diverse than that; for example, some bacterial cells are deprived of any cell wall, and some bacteria exhibit an outer mycomembrane or an outer toga [102]. Nonetheless, the different types of cell envelope play a key role in osmotic protection of the bacterial cell. In Gram-negative bacteria, lipopolysaccharide (LPS) is a major component of the outer membrane, which can limit the polyphenol connections to the peptidoglycan layers [56,103]. Cloudberry and raspberry phenolic extracts (1 mg/mL) were further reported to disintegrate the outer membrane of all examined *Salmonella* strains [87]. The bacterial outer membrane appeared to be complexed with divalent cation from the extracts and intercalated into the outer membrane and replaced by stabilizing cations, leading to membrane disintegration. Phenolic extracts of cloudberries, raspberries, and strawberries rich in ellagitannins were accountable for strong antibacterial activity by causing outer membrane disintegration, LPS release, and an increase in cytoplasmic membrane permeability [87].

The interaction between essential oils and the bacterial cell wall resulted in a change in cell envelope permeability, leading to a disturbance in the ion exchange process through the envelope, resulting in inhibition of metabolic activity in the bacterial cell [103]. The hydrophobic molecules in citrus essential oils can segregate into the bacterial membrane, resulting in membrane disruption, membrane expansion, increased membrane fluidity and permeability, loss of cytoplasmic material, inhibition of respiration and alteration of ion transports [27,89]. Essential oil components also act on cell proteins embedded in the cytoplasmic membrane by distorting lipid–protein interaction or directly affecting hydrophobic protein regions [104]. Essential oils function as membrane-activated disinfectants, which denature proteins and disrupt the outer membrane, leading to K^+^ leakage, respiration inhibition and cell lysis [105]. In addition, the penetration of essential oils into the bacterial membranes led to the leakage of ions and cytoplasmic content, which caused cell lysis and bacterial death [106]. Besides the growth inhibition observed by scanning electron microscopy, mandarin (*Citrus reticulata* L.) essential oil at 0.5–1 mg/mL demonstrated damage of *S. aureus* on the bacterial cell surface, which displayed obvious distortion with an unclear profile and a collapsed and pitted surface. Thus, the disruption of the bacterial cell membrane led to the leakage of cytoplasmic content, including proteins and nucleic acids [88]. The hydrophobicity of the cell surface and the membrane permeability of *S. aureus* were reduced by vitexin at the sub-MIC dose of 126 µg/mL [107].

Cytoplasmic pH is an essential facet of bacterial cell physiology. Cytoplasmic pH is controlled by regulation of the permeability of protons to cell membranes [108]. Upon external chemical modification, including pH changes, the cell membrane structure can be altered by changes in phospholipids and proteins [23]. The intracellular activities, e.g., DNA replication, enzymatic reactions, nutrient transport systems, flagellar synthesis and rotation, and protein synthesis, can be modified and altered when an undissociated form of organic acid diffuses across the cell membrane and lowers the intracellular pH, following dissociation of the acid hydrogen ions [109,110]. Various reports reveal the phytochemical interactions with bacterial membranes. The pH of berry fruit is in the range of 3–4 due to the presence of weak organic acids. These acids can cross bacterial cell membranes more actively than strong acids because of varying pKa values (pH of equilibrium between the undissociated and dissociated forms) [111]. Undissociated acids can freely diffuse across hydrophobic membranes [112]. Natural extract derived from *C. paradisi* (grapefruit), *C. reticulata* (mandarin), *C. aurantium* subsp. *bergamia* (bergamot) and *C. sinensis* (sweet orange), and produced in commercial form, called BIOLL, are all rich in citric acid [89]. The extracts can inhibit the growth of *S. enterica, E. coli* and *Brachyspira hyodysenteriae* at incredibly low concentrations (ranging from 20 to 80 ppm) and lead to leakage of intracellular material after 90 min of treatment, reflecting the loss of membrane integrity. The main cellular target is most likely the membrane, because citrus fruit extract can modify the carboxylic groups of membrane fatty acids.

Lee et al. [90] investigated the antimicrobial effects of various flavonoids on *E. coli* O157:H7 cell growth and found that quercetin (a flavonol) and hesperetin (a flavanone) at a concentration of 200 µM affect the morphology of *E. coli* O157:H7 by disrupting the membrane integrity, considering the observed loss of electron-dense cellular material. Furthermore, quercetin increases the cytoplasmic membrane permeability of *S. pyogenes*, resulting in a bacteriostatic effect on Gram-positive bacteria at the MIC of 128 μg/mL [91]. Stern et al. [113] found that phenolic compounds in low pH conditions may complex with proteins on the microbial outer membranes through non-specific forces, e.g., hydrogen bonding and hydrophobic effects, covalent bond formation and changed Na^+^/H^+^ antiporter systems, to reduce bacteria tolerance in low osmotic environments. These interactions result in bacterial death [114].

### 3.2. Effects on Cell–Cell Communication and Gene Regulation

Communication between bacterial cells by extracellular biochemical signals is an important molecular mechanism in bacterial physiology, ecophysiology and physiopathology. Cell–cell communication through signal molecules plays a key role in sessile development and biofilm formation for bacteria to adapt to different microenvironments and access superior nutrients, and also in community behavior between planktonic and sessile cells [115]. Of note, when cell–cell communication specifically refers to probing the number of cells in a population in order to adapt its physiological response, it is then defined as quorum sensing (QS) [56]. The signaling molecules participating in bacterial cell–cell communication are small, diffusible and functional molecules called autoinducers (AI) [93]. Type I autoinducers (AI-1) are involved in intra-specific communication in Gram-positive or Gram-negative bacteria, whereas AI-2 are involved in inter-specific communication among both Gram-positive and Gram-negative bacteria [93].

Different flavonoids from citrus, especially naringenin, quercetin, sinensetin and apigenin, emerged as potent, nonspecific inhibitors of AI-mediated cell–cell signaling in pathogenic bacteria [92]. In grapefruit extract, furocoumarins (100 µg/mL) which are polyphenol-derived phototoxic compounds, have been shown to inhibit the activities of both AI-1, i.e., oligopeptides and N-acylhomoserine lactones (AHL), and AI-2, i.e., boronated-diester molecules, by more than 95% in *E. coli* O157:H7, *S*. Typhimurium and *P. aeruginosa* [93]. Limonoids, constituents of grapefruits and other citrus fruits, demonstrated the inhibition of AHL (developed AHL) and AI-2 mediated cell–cell signaling, and resulted in a decrease in cell–cell communication and biofilm formation, as well as the repression of the expression of Type III secretion system (T3SS) in enterohemorrhagic *E. coli* (EHEC) [94]. Grape seed extract, a by-product of the wine industry, at concentrations as low as 0.5 mg/mL, could reduce AI-2 production by all non-O157 STEC (Shiga toxin-encoding *E. coli*) tested, and could reduce production of the flagellum protein (FliC) and its regulator (FliA) in *E. coli* O103:H2 and *E. coli* O111:H2. Moreover, such extracts inhibited the production of the Shiga toxin (Stx), the major virulence factor of STEC, in both strains [95]. Malic, lactic, and acetic acids have been reported to harbor anti-QS activity for *E. coli* and *Salmonella* sp., with a higher potential than citric acid [116]. At concentrations of 1–4%, malic acid and lactic acid have a negative effect on AI-2 activities in *E. coli* O157:H7 and *S.* Typhimurium [117]. Malic acid was effective in the complete inhibition of *S.* Typhimurium surface biofilm [118]. Lactic acid at a concentration of 1% was found to reduce the production of extracellular polymeric substances (EPS) by 13%, whereas citric acid and acetic acid exhibited a 6% and 11% reduction, respectively [116].

Citral and limonene essential oils revealed antibiofilm activity against planktonic cells of several bacterial pathogenic species, including MSSA, MRSA, VRE, VSE, *L. monocytogenes* and *E. coli* [119,120,121]. Baicalein, a derivative of flavones, at concentrations of 32–64 μg/mL was shown to downregulate the QS regulators *agrA*, *sarA* and RNAIII, as well as the intercellular adhesin (*ica*) gene cluster in *S. aureus* biofilm producer cells [122]. Vitexin (500 µg/mL) is a flavone that downregulates *icaAB* and *agrAC* gene expression, resulting in antibiofilm and bactericidal effects on *E. coli* and *P. aeruginosa* [25]. Triterpenoid acids also inhibit *agr*-mediated QS and decrease δ-toxin production and biofilm formation, with weak or no inhibition of planktonic growth of *S. aureus* [96]. Triterpenoid acids from pink peppercorn (*Schinus terebinthifolia,* IC_50_ 2–70 μM) inhibit all *S. aureus agr* alleles, which have a regulatory role in the virulence of MRSA [96]. Triterpenoid acids also inhibit other *agr*-regulated reporters in a dose-dependent manner, such as leucocidin A (*lukA*, IC50 0.4–25 μM) and glycerol ester hydrolase or lipase (*gehB*, IC50 1.5–25 μM) [96]. Ellagic acid, the major component of methanol pomegranate (*Punica granatum* L.) extract, was proposed to interfere with QS, leading to antibiofilm activity by disrupting pre-formed biofilms of MRSA, MSSA and *E. coli* at a concentration of 20–40 µg/mL [123].

Besides inhibiting the growth of *Vibrio cholerae* at the MIC of 125 g/L, red bayberry extract (*Myrica rubra*) could repress virulence genes encoding the cholera toxin (CT) and the toxin-coregulated pilus (TCP) [124]. While CT is well-known as the causative agent of cholera, leading to severe diarrhea, TCP has a key role in the early attachment of vibrios to the intestinal epithelium and is required for intestinal colonization [125]. Citrus peel extracts are rich in bioactive compounds, such as saponins, tannins, flavonoids, steroids, and alkaloids, which were further shown to reduce the expression of *ldh*, *toxA* and *toxB* in *V. parahaemolyticus*.

### 3.3. Inhibition of Metabolic and Enzyme Activities

By testing the effects of diverse polyphenols on c-di-AMP synthase from *B. subtilis*, it appeared that theaflavin-3′-gallate and theaflavin-3,3′-digallate exhibited inhibitory effects on the enzymatic activity [126]. In a similar approach, various tannins and polyphenolic compounds were identified as activity inhibitors of NADH-ubiquinone-1 oxidoreductase in *Paracoccus denitrificans*, *B. subtilis*, *Photobacterium phosphoreum* and *Thermus thermophilus* [97]. The precursors of ellagitannins, pentagalloylglucose, gallotannin, sanguiin H-11 and oolonghomobisflavan A, as well as polymerized procyanidin, were shown to be proficient inhibitors of NADH dehydrogenases (NDH) [98]. In general, condensed tannins are considered as inhibitors of extracellular microbial enzyme activity as well as microbial growth and metabolism [85]. Flavonoid compounds present in cocoa were shown to be mild inhibitors of cyclic AMP-stimulated chloride ion (Cl^−^)-secretion in colonic epithelial cells [14,127].

Phytochemicals in fruits can inhibit the activity of bacterial toxins. Fruit extracts of Chinese quince (*Chaenomeles speciosa*) with the IC_50_ value of 193.1 µg/mL were reported to inhibit *E. coli* heat-labile toxin (LT)-induced diarrhea in mice by disposing the binding of the B subunit of LT (LTB) to G_M1_ [99]. Gallic acid present in the peel extract of *Campomanesia adamantium* at 10 μM significantly decreased the levels of cGMP-stimulated *E. coli* enterotoxin in human colorectal carcinoma cells (T84 cells) [128]. Fruit extracts of *Prosopis alba* Griseb. and *Ziziphus mistol* Griseb. are able to inhibit the toxic action of Stx from EHEC [129]. Grape extract inhibits the cholera toxin (CT), a heterohexameric AB_5_ protein complex produced by *V. cholerae* and LT in *E. coli* [100]. Several individual polyphenolic compounds (>25 μg/mL) from grape extract could protect cultured cells from specific toxins or subsets of toxins [130]. Caftaric acid acted as an inhibitor of CT and the diphtheria toxin (DT) from *Corynebacterium diphtheriae.* Epicatechin gallate was shown to act as an inhibitor of exotoxin A (ETA) from *P. aeruginosa*, whereas epigallocatechin gallate (EGCG) was an inhibitor of DT, CT, and ETA [130]. Applephenon, a polyphenol derived from apple, at a concentration of 200 µg/mL, was also reported to inhibit the ADP-ribosyltransferase activity of the cholera toxin (CT) and consequently inhibit fluid accumulation in mouse ileal loops [101].

### 3.4. Fruit Extracts: Natural Antibiotics against Multi-Resistant Bacteria

Antimicrobial resistance (AMR) is one of the top 10 global public health threats [131]. The misuse and overuse of antibiotics are the main drivers in the progression of multi-drug resistance (MDR) species that increase the complexity and severity of the disease [14]. β-lactams are one of the largest classes of antibiotics. Bacteria have developed resistance to every β-lactam antibiotic used. The bacterial resistance mainly occurs by the production of β-lactamases [132]. A study of *Terminalia ferdinandiana*, an Australian fruit, demonstrated that it has strong antibacterial activity against β-lactam-sensitive and -resistant *E. coli* strains, and is active against methicillin-sensitive and -resistant *S. aureus* with MIC values of 388–9250 µg/mL. The tannin and flavonoid content in *T. ferdinandiana* extract inhibit bacterial pathogens through different mechanisms such as bacterial cell wall disruption, binding to cell surface proteins and inhibiting bacterial enzymes [132]. An Indian species, *Terminalia belliricia* (Gaertn.) Roxb., has also been studied. The aqueous and methanol extracts of *T. bellirica* showed antibacterial activity against MDR bacteria (i.e., MRSA, extended spectrum β-lactamase producing *E. coli*, MDR *Acinetobacter* spp. and MDR *P. aeruginosa*) with MIC values of 0.25 to 4 mg/mL [133]. Borojó (*Borojoa patinoi* Cuatrec.) aqueous extract (BAE) at concentrations of 187–375 mg/mL was effective against the strains of *P. aeruginosa* with a high multidrug resistance (e.g., β-lactams, quinolones, chloramphenicol, tetracycline, macrolides, trimethoprim-sulfamethoxazole and rifampin) [134]. BAE also contains phenols, which increase membrane permeability, leading to the loss of cellular material and, ultimately, bacterial death. In studying the native plants of Chile, fruits of arrayan (*Luma apiculata* (DC.) Burret) and peumo (*Cryptocarya alba* (Molina) Looser), only the peumo extract showed an inhibitory effect against drug-resistant strains of *S. aureus* and *P. aeruginosa,* with IC_50_ of 0.55 and 0.78 mg/mL, respectively. The arrayan extract was effective in inhibiting the adhesion of drug-sensitive *S. aureus* and *P. aeruginosa* with an IC_50_ of 0.23 and 0.29 mg/mL [135], respectively. Both extracts also inhibited the autoinducer-2, a quorum sensing signal that mediates communication, in a concentration-dependent manner. The peel extract of *P. granatum* Linn. (pomegranate) exhibited antimycobacterial activity against a broad panel of *Mycobacterium tuberculosis* and β-lactamase-producing *K. pneumoniae* isolates with MIC values of 256–1024 μg/mL [136]. Epigallocatechin-3-gallate (EGCG) and quercetin, a polyphenolic compound in pomegranate, were able to inhibit *M. tuberculosis* and *K. pneumoniae* isolates with MIC values of 32–256 µg/mL. This compound inhibited DNA gyrase by binding to the ATP binding site of the gyrase B subunit [136].

### 3.5. Fruit Extracts and Gut Microbiota

Polyphenol compounds and gut microbiota have different ways of interaction. Ellagitannins from pomegranates, anthocyanins, and resveratrol significantly enhanced the growth of beneficial bacteria such as *Bifidobacterium* spp., *Lactobacillus* spp. and *Enterococcus* spp. [137,138,139]. These compounds may induce positive modulation of gut microbiota. These gut microbiota play an essential role in promoting human health. Many types of fruit polyphenols have very low bioavailability; therefore, they reach the colon in an unchanged form. The bioavailability is related to chemical structure [140]. For example, aglycones, simple polyphenols that are absorbed by the intestinal mucosa in the small intestine; in turn, glycosides and complex structure are only partially absorbed by the small intestine, and most of them reach the colon [141]. These events occur similarly to the polyphenolic polymer form. In the colon, the gut microbes can transform the polyphenols into bioactive metabolites and influence the microbial ecology, which can improve both gut health and overall health status [140,142].

## 4. Antioxidant Properties of Fruit Extracts

Polyphenols are generally considered as the main group of antioxidant molecules that function through different mechanisms, such as the suppression of free radicals that initiate oxidative damage and inhibit the oxidation process via chelation of catalytic metals or metal ions, and inhibition of fruit lipoxygenase [143,144].

Antioxidant capacity is associated with chemical compounds that can protect biological systems from potentially harmful effects of processes or reactions involving reactive oxygen and nitrogen species [145]. The total antioxidant capacity of fruit extracts can be described in different assays, including Trolox equivalent antioxidant capacity (TEAC), oxygen radical absorbance capacity (ORAC) assay, ferric reducing ability of plasma (FRAP), and cupric reducing antioxidant capacity (CUPRAC). TEAC, FRAP and CUPRAC are spectrophotometric, whereas ORAC is a fluorometric assay [146]. Study of the antioxidant capacity of four Brazilian native fruits, araçá (*Psidium cattleianum*), butiá (*Butia eriospatha*), pitanga (*Eugenia uniflora*) and blackberry (*Rubus* sp.), found that xavante blackberries and purple-fleshed pitanga contained the highest total phenolic content (359–816 mg gallic acid equivalent/100 g fresh weight), including quercetin derivatives, quercitrin, isoquercitrin, and cyanidin derivatives [147]. Purple-fleshed pitanga had the highest scavenging activity in the 2,2-diphenyl-2-picrylhydrazyl hydrate (DPPH) assay, with IC_50_ of 37 mg/L, and also showed the highest FRAP, followed by xavante blackberries, Cherokee blackberries, and araçá. Moreover, bioactive compounds from peel of a Brazilian native fruit, umbu fruit (Spondias tuberosa), showed total phenolic compounds (1985 mg gallic acid equivalent/100 g), total flavonoid compounds (1364 mg rutin equivalent/100 g) and antioxidant capacity by ABTS (122 µmol Trolox equivalent/g), DPPH (174 µmol/Trolox equivalent g) and FRAP assays (468 µmol Fe^2+^/g) [148]. It can be concluded that fruits rich in phenolic compounds have high antioxidant activity.

The edible wild fruits of 20 plant species were reported on, where *Prunus domestica* (plum) and *Rubus ellipticus* fruits had the highest levels of phenolics and flavonols, i.e., 113 mg gallic acid equivalent/100 g fresh weight of sample (mg GAE/100 g, FW) and 200 mg rutin equivalent per 100 g of fresh sample (mg RtE/100 g), respectively [149]. Extracts of *Rosa moschata* exhibited the highest amount of flavonoids with 194 mg RtE/100 g FW, whereas *Duchesnea indica* fruit showed significant potential to scavenge DPPH at 83.54%. Additionally, the total antioxidant capacity was the highest in the extract of *Berberis lycium* fruit with 332 µM ascorbic acid equivalent per 100 g based on fresh weight (AAE/100 g FW) [149]. Among the methanolic extracts of ten fruits, jambul and acerola presented the greatest values of antioxidant capacity by DPPH assay with IC_50_ of 21 and 24 µg/mL and total phenolic compounds of 635 and 675 mg gallic acid equivalent/100 g of sample, respectively [150]. This result showed a positive correlation between the antioxidant capacity and total phenolic content, indicating that phenolic compounds may be the major components involved in the inhibition of free radicals in fruits. The total antioxidant capacity was high in various berries such as blueberries, blackberries, bilberries, raspberries and black currants, as well as chokeberries (*A. melanocarpa*) [151]. Blueberries, with a TEAC value of 15 mM Trolox/100 g dry weight (DW), exhibited the strongest total antioxidant capacity using both the 2,2-azinobis (3-ethylbenzothiazoline-6-sulfonic acid) diammonium salt (ABTS) and the DPPH methods. Blueberries also had the highest total phenolic content (9 mg gallic acid/g DW), total flavonoid content (36 mg rutin/g DW), and total anthocyanidin content (24 mg catechin/g DW) [152]. The immature wild blueberry (*Vaccinium stenophyllum* Steud.) fruit extract had the highest content of total phenolic compound (19.153 ± 0.175 mg gallic acid equivalent /g DW) and the highest antioxidant activity by ABTS (196.761 ± 0.641 µM Trolox equivalent /g DW) and DPPH assays (146.580 ± 6.466 µM Trolox equivalent /g DW), whereas mature blueberry fruit extract exhibited the highest content of anthocyanins (0.141 ± 0.004 mg cyanidin-3-glucoside equivalent/g DW) and cyanidin-3-glucoside (19.230 ± 0.309 mg cyanidin-3-glucoside equivalents/g DW) [153]. The concentration of phenolic compounds and anthocyanins in aronia berries is approximately 2080 mg/100 g of fruit and 3529 mg/L, respectively, which is higher than other berries. The antioxidant capacity of aronia berries is also the highest (159 μM of Trolox equivalent/g fresh weight measured by ORAC assay) [154]. In small berries (blackberries, black currants, blueberries, goji berries, raspberries, red currants, red gooseberries, white currants, and white gooseberries), the highest antioxidant capacity was recorded in black currants due to their high anthocyanin content [155]. Black currant anthocyanins, dosed at 8 and 6 mg of cyanidin-3-glucoside equivalent/g in extracts and fresh material, respectively, demonstrated a high antioxidant capacity using TEAC (12 mM Trolox equivalent/g extract and 9 Trolox equivalent/g fresh matter), FRAP (10 mM Fe^2+^ equivalent/g extract and 7.72 Fe^2+^ equivalent/g fresh matter), and DPPH (IC_50_ 0.20 mg/mL) assays [155].

### 4.1. Free Radical Scavenging Activity

Free radicals can be defined as any atoms or molecules with unpaired electrons. These unstable and highly reactive species can donate electrons to or receive hydrogen atoms from other molecules, thus behaving as reductants or oxidants [156]. A stable antioxidant molecule can donate hydrogen or electrons to a free radical and neutralize it. In studying the free radical scavenging capacity and the antioxidant activity of plums (*P. domestica* L.), it appears that fresh plum is more effective in collecting oxygen free radicals, such as superoxide (O_2_^−^) and peroxy radicals (ROO•), than dried plum extract, which has a maximum nitric oxide (NO) radical scavenging activity [157]. As a consequence of higher concentrations of phenolic (625.93 ± 14.08 mg GAE/100 g extract) and flavonoid compounds (35.81 ± 0.47 mg quercetin equivalent/100 g extract), the antioxidant capacity (16.64 ± 0.58 mg α-tocopherol/g extract) of dried plum was also higher than that of fresh plum. The higher concentrations of phenolic acids and flavonoids in dried plum also highly correlated with the reducing power, chain-breaking antioxidant activity, the quantity of malondialdehyde, as well as DPPH and NO radical scavenging activities [157]. Hydroxycinnamic acids can be described as chain-breaking antioxidants acting through radical scavenging activity, which is related to their ability to exchange electrons and delocalize or stabilize phenoxyl radicals [67a]. The phytochemicals responsible for antioxidant abilities in fruits are mainly due to phenolic acids and flavonoid compounds. The main structural properties responsible for the antioxidant and free radical scavenging activity are the number and position of hydroxyl groups present in the molecule [67a]. The antioxidant activity of phenolic compounds is related to the aromatic ring substitutions and the side chain structure [143]. In studying 16 phenolic compounds differing in their patterns of hydroxylation and methoxylation on their aromatic rings, it appeared that phenolic compounds with multiple hydroxyl groups (-OH) (e.g., protocatechuic acid, pyrogallol, caffeic acid, gallic acid and propyl gallate) presented higher free radical scavenging activity than monohydroxylated aromatic cycles (e.g., p-coumaric acid and ferulic acid) (Table 2), particularly against DPPH^•^ and O_2_^•−^ [158]. The number of hydroxyl groups has been shown to be crucial in determining the antioxidant properties [159]. As a matter of fact, trihydroxylated (pyrogallol moiety) phenolic acids displayed higher antioxidant activity than dihydroxylated (catechol moiety) ones. Hydroxycinnamic acid derivatives are more effective antioxidants than benzoic acid derivatives, the latter showing higher activity than aldehyde and alcohol derivatives of benzene [158]. However, in flavonoid compounds, the position of the hydroxyl groups on the aromatic ring is more important than their number [56a]. Baicalin, flavonoid ortho-dihydroxyl groups on ring A (in the 5, 6 positions), had a stronger OH-scavenging activity than lysionotin and matteucinol, flavonoid meta-dihydroxyl on ring A (in the 5, 7 positions) [160]. In addition, flavonoid hydroxyl groups in the 3′, 4′ positions of ring B such as guercetin, heliosin, hyperoside, baicalin, lysionotin, and matteucinol, possessed higher OH-scavenging activity than the flavonoid hydroxyl groups on ring A.

### 4.2. Complexation of Metal Ions

**Figure 1:**
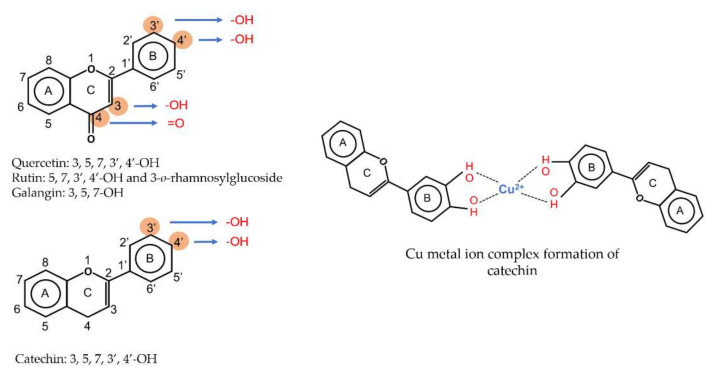
Metal ion complexation from flavonoids. Flavonoids that have ability to chelate metal ions (Fe^3+^, Cu^2+^) at the sites include 3′,4′-dihydroxy group on the B ring (rutin and catechin) and 3-hydroxy and 4-oxo groups on the C ring (galangin and quercetin).

The transition metal ions are able to function in oxidation states. Transition metal ion chelators, or metal ion complexation, can control oxidation [56a]. In investigating the natural transition metal coordination anthocyanin complex as free radical scavenger in java plum (*Syzygium cumini*), cyanidin-3,5-*O*-diglucoside was revealed to act as a chelator with a catechol structure on the B ring [161]. Cyanidin can act as a chelator of various metal ions such as Fe^2+^, Co^2+^, Ni^2+^, Cu^2+^ and Zn^2+^. The sites in flavonoid and anthocyanin molecules that can interact with metal ions include 3′,4′-dihydroxy group on the B ring, 3-hydroxy or 5-hydroxy, and the 4-carbonyl groups on the C ring [161,162]. As a ligand, cyanidin strongly stabilizes Fe^3+^ over Fe^2+^. The complexes of Fe^2+^, catecholate and gallate, are immediately oxidized by O_2_ to form the Fe^3+^-polyphenol complexes. Chelation of Fe^2+^ leads to an electron transfer reaction in oxygen, producing the Fe^3+^-polyphenol complex and the development of an extra stable complex. Flavonoids displayed antioxidant activity through chelating with transition metals, primarily Fe (II), Fe (III) and Cu (II), which are involved in free radical-generating reactions. Complexations of metal flavonoids or metal-flavonoid chelates are stronger free radical scavengers than free flavonoids. These play key roles in protection from oxidative stress [162]. The formation of complexes of metal ions with the flavonoids quercetin, rutin, galangin and catechin were investigated with UV-visible spectroscopy and indicated that the interactions of Cu(II), Fe(II), Al(III) and Zn(II) ions with quercetin at a 2:1 (metal: flavonoid) ratio operated in bathochromic shifts in the absorption bands in the UV/visible region [163]. The first sites involved in the complexation process are the acidic proton and 3-hydroxy groups such as 3-OH and 4-oxo groups, followed by the 3′,4′-dihydroxy groups. Additionally, metal ions were bound exclusively to 3′,4′-dihydroxy groups (rutin and catechin) and to 3-OH and 4-oxo groups (galangin and quercetin) (Figure 1). The pH also impacts the complex formation of a flavonoid. Complexes with the highest coordination numbers usually occur in slightly acidic or neutral pH. The optimal pH for complex formation is around pH 6 [162].

### 4.3. Lipoxygenase Inhibition

Lipoxygenase (LOX) is an enzyme that catalyzes the dioxygeneration of polyunsaturated fatty acids with a cis, cis-1,4-pentadiene unit to form conjugated hydroperoxydienoic acids [164]. Lipoxygenase can affect fruit growth, pest resistance and senescence but has negative implications for color, flavor and antioxidant ability. The different lipoxygenases catalyze oxygen at different positional specificities along the carbon chain, known as regiospecificity [165]. This specificity has significant effects in the resultant hydroperoxide metabolism on the quantity of secondary metabolites [164]. Lipoxygenase catalyzes the peroxidation of free linoleic acid or linolenic acid, leading to the disintegration of hydroperoxide through a reaction catalyzed by the hydroperoxide lyase. The degradation of these polyunsaturated fatty acids through the lipoxygenase pathway generates the aroma of fruits and vegetables [166]. Oxygen insertion takes place at position 9 or 12 to form the corresponding 9 or 13 hydroperoxide [164]. Fruit polyphenols can inhibit lipoxygenase activity through three mechanisms [56a]: (i) the ability to bind to a hydrophobic active site, (ii) lipid radical scavenging, and (iii) interaction with the hydrophobic fatty acid substrate. At 0.5 mg/mL, the methanol and chloroform extracts of *Gaultheria trichophylla* (Royle), commonly known as Himalayan snowberry, showed the strongest inhibition of the 5-lipoxygenase enzyme, of 90.5% and 66.9%, and with IC_50_ values of 277.3 µg/mL and 379.5 µg/mL, respectively [167]. Methanol extracts of *Cydonia oblonga* (quince fruit) strongly inhibited lipoxygenase with IC_50_ values of 99.3 µg/mL, and other extracts (water, ethyl acetate, chloroform, and butanol) also notably inhibited lipoxygenase with IC_50_ values in the range of 101.8 to 227.3 µg/mL [168]. The inhibitory activity of 2-(3,4-dihydroxyphenyl) ethanol from olive fruit extract was found present in both 12-lipoxygenase (12-LO) and 5-lipoxygenase (5-LO) of the arachidonate lipoxygenase activities [169]. *Rubus idaeus* L. (Polana raspberry) fruit and juice had the highest ability to inhibit the activity of lipoxygenase with 0.8 and 0.6 mg fresh weight (FW)/mL, respectively. In contrast, anthocyanin-rich fractions had the lowest ability to inhibit lipoxygenase with an IC_50_ of 4.5 mg FW/mL [170].

## 5. Product Development from Fruit Extracts

Fruits are known for their significant antioxidant activity due to their high content of polyphenolic compounds. Fruit beverages are products in high demand on the market due to their sensory properties and health-promoting value. Among homemade whey beverages, market whey beverage and fruit mousses, the red fruit mousse (apple 58%, banana 25%, cherry 9%, beetroot 8%) demonstrated the highest content of polyphenolic compounds (76.41 mg/100 g). Moreover, the green fruit mousse (apple 65%, zucchini 15%, banana 10%, spinach 5%, kiwi 5%) contained the highest content of flavonoids (69.80 mg/100 g) [171]. Antioxidants of fruit extracts have been applied to biomedical and biotechnological applications including medicine, food, animal feed, cosmetic substances, and pharmaceuticals. *Carica papaya* fruit extract contained active ingredients that could be competitive reducing and stabilizing agents during phytofabrication of nanoparticles. Therefore, the development of selenium nanoparticles (SeNPs) from *C. papaya* extract has been promoted to suppress microbial pathogen and cancer-cell proliferation [172]. SeNPs were shown to be non-toxic at low levels (25 and 50 μg/mL) and did not cause *Danio rerio* embryo death at lower concentrations in vivo. However, detailed toxicological trials should be established to clarify their safety for practical use [172]. In meat industries, lemon peel powders (1%), with the highest levels of bioactive substances (90.5 mg gallic acid/g total phenolics and 35 mg rutin/g total flavonoids), could be used in the manufacture of meat products as natural additives to improve microbial quality and shelf life by delaying microbial growth [173].

## 6. Conclusions and Perspective

Antibiotics have been widely used to suppress pathogen infections for more than 70 years. Within the last few decades, however, bacteria resistant to multiple antibiotics have become increasingly widespread, leading to difficulties in controlling and curing some infectious diseases in humans. In this context, interest in plant phytochemicals as alternative treatment strategies in bacterial infections has increased. Fruit extracts consist of a combination of functional components, including bioactive antioxidant compounds. Several antimicrobial mechanisms within these substances have been described, and our knowledge of their bioactivity to treat and prevent human diseases has improved. Using fruit extracts as an antibiotic alternative is attractive, considering that the development of bacterial resistance to a mixture of active molecules may be slower than acting on a single compound, as generally used in antibiotic therapy [14], and that fruit extracts could also preserve probiotic species in the microbiota [87a]. Nonetheless, further studies are still required and should be conducted to clarify the mechanisms of action of phytochemicals in such mixtures. Furthermore, the efficacy of phytochemicals is mostly tested and evaluated in simple in vitro models, and the results can be quite different from the complex situations encountered in the gastrointestinal tract. Besides experimental testing in animal models, the use of artificial digestive models should be evaluated and proved their efficiency in human health [174,175].

## Figures and Tables

**Table 1:** Components of fruit extracts as antimicrobial agents.

Extract/Compound	Major Fruit Source	Antimicrobial Action	Inhibitory Concentration	References
**Organic acids**				
Citric acid	Citrus fruits	*S. aureus, S. dysenteriae, E. coli* and *P. aeruginosa*	Plate count method, 1–6%	[20,21]
Malic acid	Apples, blackberries, cherries, apricots, peaches, mangos, and plums	*L. monocytogenes, E. coli* O157:H7 and *S.* Gaminara	Antibacterial effectiveness on film disc, 2.6%	[22]
Propionic acid	Apples and strawberries	*S. aureus, S. lutea, L. monocytogenes* and *P. vulgaris*	Growth inhibition, 0.3%	[23]
Tartaric acid	Grapes	*L. monocytogenes, S.* Gaminara and *E. coli* O157:H7	Antibacterial effectiveness on film disc, 1.8–2.6%	[22]
Tartaric acid, citric acid and acetic acid	Cranberries, bilberries, blueberries, blackberries, raspberries, black chokeberries, red currants, and blackcurrants	*S.* Typhimurium	MIC, 0.312–1.25%	[24]
Salicylic acid	Berry fruits	*S. aureus, E. coli, P. aeruginosa* and *E. faecalis*	MIC, 250–500 µg/mL	[25]
**Terpenes**				
Eugenol	Strawberries, blackberries, bananas, and citrus	*S.* Typhimurium	MIC, 0.07 mg/mL	[26]
Terpineol	Apples, blueberries, and limes	*S. aureus*	MIC, 0.12 mg/mL	[26]
Carveol, citronellol and geraniol	Citrus fruits	*E. coli*	MIC, 0.25 mg/mL	[26]
Thymol, (+) menthol, and linalyl acetate	Bergamot	*S. aureus* and *E. coli*	MIC, 0.31–1.25 mg/mL	[27]
**Flavonoid polyphenols**				
Flavones	Oranges, grapefruits, and lemons	*E. faecalis, E. coli,* and *P. aeruginosa*	MIC, 500 µg/mL	[25]
Flavonols	Berries, black grapes, apricots and apples	Gram-positive (*S. aureus,* MRSA, *S. epidermidis*, *S. haemolyticus,* and *S. pyogenes*) Gram-negative (*E. coli, S.* Typhimurium and *K. pneumoniae*)	MIC, 0.25 mg/mL	[28]
Flavan-3-ols (3′-O-methyldiplacol)	Apples, grapes, blackberries, and cranberries	Gram-positive bacteria (*B. subtilis, E. faecalis, L. monocytogenes, S. aureus,* and *S. epidermidis*)	MIC, 2–4 μg/mL	[29]
Flavan-3-ols (Quercetin 3-O-methyl ether)	Apples, grapes, blackberries, and cranberries	*H. pylori*	MIC, 3.9 µg/mL	[28]
Flavanones	Citrus fruits	Gram-positive bacteria (*B. cereus, B. subtilis, E. faecalis, L. monocytogenes,* and *S. aureus*)	MIC, 2–4 μg/mL	[29]
Flavanones (Sophoraflavanone G)	Citrus fruits	*S. epidermidis, S. aureus,* and *B. subtilis*	MIC, 3.1–12.5 μg/mL	[28]
Anthocyanins	Grapes, cherries, plums, raspberries, strawberries, blackberries, apples, peaches, red and blackcurrants, blueberries, and cranberries	*E. coli* and *Salmonella* sp.	MIC, 10–400 mg/mL	[30]
Anthocyanins	Blueberry extract	*S. aureus* and *E. coli*	Biofilm formation, 500 µg/mL	[31]
	**Non-flavonoid polyphenols**			
Phenolic acids	Blueberries and muscadines	*S.* Enteritidis	Growth inhibition, 24–46 µg/mL	[32]
Phenolic acids	Cranberries	*L. monocytogenes*	Plate count method, 10 mg/mL	[10]
Phenolic acids	Cranberry extract	*S. oralis, A. naeslundii, V. parvula, F. nucleatum, P. gingivalis* and *A. actinomycetemcomitans*	Antibiofilm activity, 0.1–1.0 mg/mL	[33]
Phenolic acids (4-hydroxybenzoic acid and trans 4-hydroxycinnamic acid)	Blueberries, cloudberries and bilberries	Gram-positive (*S. aureus, S. epidermidis*, and *B. subtilis*) Gram-negative (*E. coli, S*. Typhimurium, *P. aeruginosa,* and *P. syringae*)	Disc diffusion method, 100–170 µg/mL	[34]
Phenolic acids (Tannic acids)	Berries, pomegranates, grapes, persimmons, pears, and apples	*S.* Typhimurium, *S.* Enteritidis, *E. coli* and *S. aureus*	MIC, 0.3–3 mg/mL	[35,36,37]
Phenolic acids (Ellagitannins)	Bilberries, lingonberries, cranberries, red raspberries, cloudberries, strawberries, blackcurrants and sea buckthorn berries	*Staphylococcus* sp.	Plate count method, 2 mg/mL	[10]
Stilbenes (Triacid derivative 135 C)	Grapes, pomegranates, bilberries, blueberries and mulberries	*S. epidermidis, S. pneumoniae, S. pyogenes, Micrococcus* spp., and MRSA	MIC, 1–32 μg/mL	[38]
Lignans (Hydroxymatairesinol (HMR) lignan)	Lingonberries, strawberries, apples, cranberries, prunes, and pears	*S. epidermidis*, *Proteus* sp., and *Klebsiella* sp.	Disc diffusion method, 25–100 mg/mL	[39]
Xanthones (α-mangostin)	Mangosteen	Gram-positive (*B. subtilis* and *S. aureus*) Gram-negative (*E. coli* and *P. aeruginosa*)	MIC, 30–100 µg/mL	[40,41]
Xanthones (*γ*-mangostin)	Mangosteen	MSSA, MRSA, VSE and VRE	MIC, 3.13–6.25 µg/mL	[42]

Note: MIC = Minimum Inhibitory Concentration.

**Table 2:** Polyphenols and their derivatives in fruit.

Polyphenols	Structure	Derivatives	Sources	References
**Flavonoids**				
Flavones	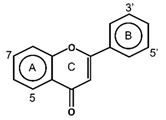	Apigenin Baicalein Chrysin Luteolin	Herbs (parsley, celery) Citrus fruits (oranges, grapefruits, and lemons)	[28,56]
Flavonols	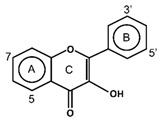	Isohanmetin Kaemferol Myricetin Quercetin Rutin	Cranberries, black currants, lingonberries, blueberries, black grapes, apricots, and apples	[9,57]
Flavan-3-ols	Monomers 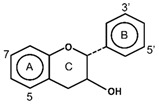 Polymers 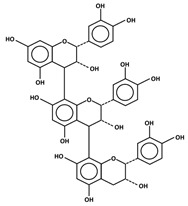 Proanthocyanidins (Condensed Tannins)	Catechin Epicatechin Gallocatechin	Apples, grapes, blackberries, and cranberries	[9,28]
		Procyanidin A2 Procyanidin B1 Procyanidin B2 Procyanidin C1	Grapes, apples, bilberries, cranberries, strawberries, and blueberries	[58]
Isoflavones	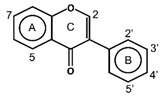	Daidzin Equol Formononetin Genestein Glycitein	Soy products, legumes, currants, and raisins	[56,59,60,61]
Flavanones	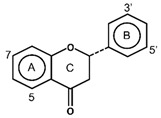	Hesperidin Narinagin Sophoraflavanone G	Prunes and citrus fruits (lemons, oranges, and grapefruits)	[9,28]
Anthocyanins	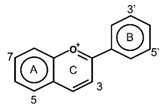	Cyanidin Delphindin Malvidin Pelagonidin Peonidin	Families Vitaceae (grapes), Rosaceae (cherries, plums, raspberries, strawberries, blackberries, apples, peaches), Saxifragaceae (red and black currants), Ericaceae (blueberries and cranberries)	[62]
**Non-Flavonoids**				
Phenolic acids	*Monomers* 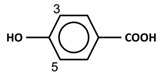 Hydroxybenzoic acids *Polymers* 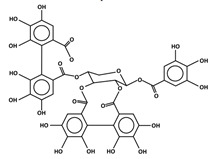 Hydrolyzable tannin (Ellagitannins)	Ellagic acid Gallic acid Protocatechuic acid Salicylic acid Syringic acid Vanillic acid	Tannic acid Genus *Rubus* (red raspberries, arctic brambles, and cloudberries), genus *Fragaria* (strawberries)	[9,10]
		Ellagitannins Gallotannins	Berries, pomegranates, grapes, persimmons, pears, and apples	[9,10,63,64,65]
Hydroxycinnamic acids	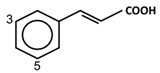	Caffeic acid Caftaric acid Chlorogenic acid Cinnamic acid Coumaric acid Curcumin Ferulic acid	Blueberries, cloudberries, and bilberries	[9,66,67]
Stilbenes	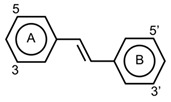	Resveratrol Piceatannol	Grape skins, pomegranates, bilberries, blueberries, and mulberries	[9,68]
Lignans	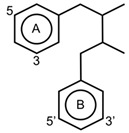	Pinoresinol Matairesinol Secoisolaricire Sinol	Strawberries, apples, cranberries, prunes, and pears	[69]
Xanthone	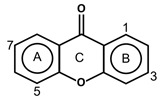	γ-mangostin	Mangosteens (*G. mangostana*)	[70]

**Table 3:** Mechanisms of action of fruit extracts as antimicrobial agents.

Antimicrobial Activity Mechanisms	Fruit Extracts or Individual Compounds	Target Bacteria	Consequences	References
Interaction with the bacterial cell envelope	Cloudberry and raspberry phenolic extracts	*S.* Typhimurium E-1151, *S.* Typhimurium SH-5014	Outer membrane disintegration, LPS release, and an increase in cytoplasmic membrane permeability	[87]
	Mandarin (*C. reticulata* L.) essential oil	*S. aureus* (ATCC 25923)	Collapse and pitting of bacterial surface cell Disruption of the cell membrane Leakage of cytoplasmic content, protein, and nucleic acid	[88]
	*C. paradisi* (grapefruit), *C. reticulata* (mandarin), *C. aurantium* subsp. *bergamia* (bergamot), *C. sinensis* (sweet orange), and BIOLL+^®^ (commercial form)	*S.* Typhimurium (CECT443, CECT883, and DT104), *S.* Enteritidis CECT4300, *S.* Infancis CECT700, *S.* Cholerasuis CECT915, *S.* London, *S.* Derby, *E. coli* (haemolytic strain), and *B. hyodysenteriae* (ATCC27164 and ATCC31212)	Membrane disruption, membrane expansion, increase in membrane fluidity and permeability, loss of cytoplasmic material, and inhibition of respiration and alteration in ion transport processes	[27,89]
	Quercetin (flavonol) and hesperetin (flavanone)	*E. coli* O157:H7 *S. pyogenes* (DMST30653, DMST30654, and DMST30655)	Disruption of membrane integrity, increased cytoplasmic membrane permeability, and loss of electron dense cellular material	[90,91]
Effects on cell-cell communication and gene regulation	Flavonoids from citrus fruits (naringenin, quercetin, sinensetin and apigenin)	*E. coli* O157:H7 ATCC 43895	Inhibition of autoinducers (AI)-mediated cell–cell signaling	[92]
	Furocumarins from grapefruit extracts	*V. harveyi* reporter strains BB886, BB170, and BB120	Inhibition of AI-1 (oligopeptides and N-acylhomoserine lactones (AHL)) and AI-2, (boronated-diester molecules)	[93]
	Limonoids	EHEC O157:H7 ATCC 43895	Inhibition of AHL and AI-2 mediated cell–cell signaling Repression of the expression of Type III secretion system (T3SS) of EHEC	[94]
	Grape seed extract	Non-O157 STEC (Shiga toxin-encoding *E. coli*)	Reduction of AI-2 production, production of flagellum protein FliC and regulator FliA in *E. coli* O103:H2 and *E. coli* O111:H2.	[95]
	Triterpenoid acids	*S. aureus*	Inhibition of accessory gene regulator, *agr*-type QS (quorum sensing), Decreased δ-toxin production and biofilm formation	[96]
Inhibition of metabolic and enzyme activities	Tannins and polyphenolic compounds	*P. denitrificans, B. subtilis, P. phosphoreum,* and *T. thermophilus*	Inhibition of activity on NADH dehydrogenases (NDH) and NADH-ubiquinone-1 oxidoreductase	[97,98]
	Chinese quince (*C. speciosa*) extract	*E. coli* BL21(DE3)pLysS strain	Inhibition of *E. coli* heat-labile toxin (LT)-induced diarrhea Elimination of the binding of the B subunit of LT (LTB) to G_M1_	[99]
	Grape extract	*V. cholerae*	Inhibition of cholera toxin (CT) and *E. coli* heat-labile toxin	[100]
	Applephenon (polyphenol from apple)	*V. cholerae*	Inhibition of ADP-ribosyltransferase activity of cholera toxin (CT)	[101]

## Abbreviations

*agr*: accessory gene regulator; AHL: N-acylhomoserine lactones; AI: autoinducers; BAE: Borojó aqueous extract; CT: cholera toxin; CUPRAC: cupric reducing antioxidant capacity; DPPH: 2,2-diphenyl-2-picrylhydrazyl hydrate; DT: diphtheria toxin; EGCG: epigallocatechin gallate; EHEC: enterohemorrhagic *Escherichia coli*; ETA: exotoxin A; FRAP: ferric reducing ability of plasma; HMR: hydroxymatairesinol; IC_50_: half-maximal inhibitory concentration; *ica*: intercellular adhesin; LPS: lipopolysaccharide; LT: heat-labile toxin; MDR: multi-drug resistance; MIC: minimum inhibitory concentration; MRSA: methicillin-resistant *Staphylococcus aureus*; MSSA: methicillin-susceptible *S. aureus*; NDH: NADH dehydrogenases; NO: nitric oxide; ORAC: oxygen radical absorbance capacity; QS: quorum sensing; STEC: Shiga toxin-encoding *E. coli*; Stx: Shiga toxin; T3SS: Type III secretion system; TCP: toxin coregulated pilus; TEAC: Trolox equivalent antioxidant capacity; UPEC: uropathogenic *E. coli*; VRE: vancomycin-resistant enterococci; VSE: vancomycin-sensitive enterococci.

## References

[1] World Health Organization (WHO) The Top 10 Causes of Death. https://www.who.int/news-room/fact-sheets/detail/the-top-10-causes-of-death.

[2] Aly R., Baron S. (2014). Microbial infections of skin and nails. Medical Microbiology.

[3] Bintsis T. (2017). Foodborne pathogens. AIMS Microbiol..

[4] Flores-Mireles A.L., Walker J.N., Caparon M., Hultgren S.J. (2015). Urinary tract infections: Epidemiology, mechanisms of infection and treatment options. Nat. Rev. Microbiol..

[5] Wilson J.W., Schurr M.J., LeBlanc C.L., Ramamurthy R., Buchanan K.L., Nickerson C.A. (2002). Mechanisms of bacterial pathogenicity. Postgrad. Med. J..

[6] Barbieri R., Coppo E., Marchese A., Daglia M., Sobarzo-Sánchez E., Nabavi S.F., Nabavi S.M. (2017). Phytochemicals for human disease: An update on plant-derived compounds antibacterial activity. Microbiol. Res..

[7] Negi P.S. (2012). Plant extracts for the control of bacterial growth: Efficacy, stability and safety issues for food application. Int. J. Food Microbiol..

[8] Oz A.T., Kafkas E., Waisundara V. (2017). Phytochemicals in fruits and vegetables. Superfood and Functional Food.

[9] Martinez K.B., Mackert J.D., McIntosh M.K., Watson R.R. (2017). Polyphenols and intestinal health. Nutrition and Functional Foods for Healthy Aging.

[10] Puupponen-Pimiä R., Nohynek L., Hartmann-Schmidlin S., Kähkönen M., Heinonen M., Määttä-Riihinen K., Oksman-Caldentey K.M. (2005). Berry phenolics selectively inhibit the growth of intestinal pathogens. J. Appl. Microbiol..

[11] Howell A.B. (2007). Bioactive compounds in cranberries and their role in prevention of urinary tract infections. Mol. Nutr. Food Res..

[12] Jepson R.G., Craig J.C. (2007). A systematic review of the evidence for cranberries and blueberries in UTI prevention. Mol. Nutr. Food Res..

[13] Hisano M., Bruschini H., Nicodemo A.C., Srougi M. (2012). Cranberries and lower urinary tract infection prevention. Clinics.

[14] Dubreuil J.D. (2020). Fruit extracts to control pathogenic *Escherichia coli*: A sweet solution. Heliyon.

[15] Schreiner M., Huyskens-Keil S. (2006). Phytochemicals in fruit and vegetables: Health promotion and postharvest elicitors. Crit. Rev. Plant Sci..

[16] Pascual-Teresa D., Moreno D.A., García-Viguera C. (2010). Flavanols and anthocyanins in cardiovascular health: A review of current evidence. Int. J. Mol. Sci..

[17] Karasawa M.M.G., Mohan C. (2018). Fruits as prospective reserves of bioactive compounds: A review. Nat. Prod. Bioprospect..

[18] Mintie C.A., Singh C.K., Ahmad N. (2020). Whole fruit phytochemicals combating skin damage and carcinogenesis. Transl. Oncol..

[19] Lacombe A., Wu V.C. (2017). The potential of berries to serve as selective inhibitors of pathogens and promoters of beneficial microorganisms. Food Qual. Saf..

[20] Oulkheir S., Ounine K., Haloui N.E.E., Attarassi B. (2015). Antimicrobial effect of citric, acetic, lactic acids and sodium nitrite against *Escherichia coli* in tryptic soy broth. J. Biol. Agric. Healthc..

[21] Lieleg O., Caldara M., Baumgärtel R., Ribbeck K. (2011). Mechanical robustness of *Pseudomonas aeruginosa* biofilms. Soft Matter..

[22] Eswaranandam S., Hettiarachchy N.S., Johnson M.G. (2004). Antimicrobial activity of citric, lactic, malic, or tartaric acids and nisin-incorporated soy protein film against *Listeria monocytogenes*, *Escherichia coli* O157:H7, and *Salmonella gaminara*. J. Food Sci..

[23] Coban H.B. (2020). Organic acids as antimicrobial food agents: Applications and microbial productions. Bioprocess Biosyst. Eng..

[24] El Baaboua A., El Maadoudi M., Bouyahya A., Belmehdi O., Kounnoun A., Zahli R., Abrini J. (2018). Evaluation of antimicrobial activity of four organic acids used in chicks feed to control *Salmonella typhimurium*: Suggestion of amendment in the search standard. Int. J. Microbiol..

[25] Adamczak A., Ożarowski M., Karpiński T.M. (2020). Antibacterial activity of some flavonoids and organic acids widely distributed in plants. J. Clin. Med..

[26] Guimarães A.C., Meireles L.M., Lemos M.F., Guimarães M.C.C., Endringer D.C., Fronza M., Scherer R. (2019). Antibacterial activity of terpenes and terpenoids present in essential oils. Molecules.

[27] Trombetta D., Castelli F., Sarpietro M.G., Venuti V., Cristani M., Daniele C., Saija A., Mazzanti G., Bisignano G. (2005). Mechanisms of antibacterial action of three monoterpenes. Antimicrob. Agents Chemother..

[28] Farhadi F., Khameneh B., Iranshahi M., Iranshahy M. (2019). Antibacterial activity of flavonoids and their structure–activity relationship: An update review. Phytother. Res..

[29] Šmejkal K., Chudík S., Kloucek P., Marek R., Cvacka J., Urbanová M., Julínek O., Kokoška L., Šlapetová T., Holubová P. (2008). Antibacterial C-geranylflavonoids from *Paulownia tomentosa* fruits. J. Nat. Prod..

[30] Ma Y., Ding S., Fei Y., Liu G., Jang H., Fang J. (2019). Antimicrobial activity of anthocyanins and catechins against foodborne pathogens *Escherichia coli* and *Salmonella*. Food Control.

[31] Silva S., Costa E.M., Mendes M., Morais R.M., Calhau C., Pintado M.M. (2016). Antimicrobial, antiadhesive and antibiofilm activity of an ethanolic, anthocyanin-rich blueberry extract purified by solid phase extraction. J. Appl. Microbiol..

[32] Park Y.J., Biswas R., Phillips R.D., Chen J. (2011). Antibacterial activities of blueberry and muscadine phenolic extracts. J. Food Sci..

[33] Sánchez M.C., Ribeiro-Vidal H., Bartolomé B., Figuero E., Moreno-Arribas M., Sanz M., Herrera D. (2020). New evidences of antibacterial effects of cranberry against periodontal pathogens. Foods.

[34] Cho J.Y., Moon J.H., Seong K.Y., Park K.H. (1998). Antimicrobial activity of 4-hydroxybenzoic acid and trans 4-hydroxycinnamic acid isolated and identified from rice hull. Biosci. Biotechnol. Biochem..

[35] Akiyama H., Fujii K., Yamasaki O., Oono T., Iwatsuki K. (2001). Antibacterial action of several tannins against *Staphylococcus aureus*. J. Antimicrob. Chemother..

[36] Widsten P., Cruz C.D., Fletcher G.C., Pajak M.A., McGhie T.K. (2014). Tannins and extracts of fruit byproducts: Antibacterial activity against foodborne bacteria and antioxidant capacity. J. Agric. Food Chem..

[37] Sanhueza L., Melo R., Montero R., Maisey K., Mendoza L., Wilkens M. (2017). Synergistic interactions between phenolic compounds identified in grape pomace extract with antibiotics of different classes against *Staphylococcus aureus* and *Escherichia coli*. PLoS ONE.

[38] Man N.Y., Knight D.R., Stewart S.G., McKinley A.J., Riley T.V., Hammer K.A. (2018). Spectrum of antibacterial activity and mode of action of a novel tris-stilbene bacteriostatic compound. Sci. Rep..

[39] Al-Ani W.M., Aziz F.M. (2013). Antimicrobial activity of hydroxymatairesinol (HMR) lignan. Iraqi J. Pharm. Sci..

[40] Ragasa C.Y., Crisostomo C.J.J., Garcia K.D.C., Shen C.C. (2010). Antimicrobial xanthones from *Garcinia mangostana* L.. Philipp. Sci..

[41] Narasimhan S., Maheshwaran S., Abu-Yousef I.A., Majdalawieh A.F., Rethavathi J., Das P.E., Poltronieri P. (2017). Anti-bacterial and anti-fungal activity of xanthones obtained via semi-synthetic modification of α-mangostin from *Garcinia mangostana*. Molecules.

[42] Dharmaratne H.R.W., Sakagami Y., Piyasena K.G.P., Thevanesam V. (2013). Antibacterial activity of xanthones from *Garcinia mangostana* (L.) and their structure–activity relationship studies. Nat. Prod. Res..

[43] Walker R.P., Famiani F. (2018). Organic acids in fruits: Metabolism, functions and contents. Hortic. Rev..

[44] Li J., Zhang C., Liu H., Liu J., Jiao Z. (2020). Profiles of sugar and organic acid of fruit juices: A comparative study and implication for authentication. J. Food Qual..

[45] Viljakainen S., Visti A., Laakso S. (2002). Concentrations of organic acids and soluble sugars in juices from Nordic berries. Acta Agric. Scand. B Soil Plant Sci..

[46] Sabra W., Dietz D., Zeng A.P. (2013). Substrate-limited co-culture for efficient production of propionic acid from flour hydrolysate. Appl. Microbiol. Biotechnol..

[47] Adamczak A., Buchwald W., Kozlowski J. (2011). Variation in the content of flavonols and main organic acids in the fruit of European cranberry (*Oxycoccus palustris* Pers.) growing in peatlands in peatlands of North-Western Poland. Herba Pol..

[48] Takita M.A., Berger I.J., Basílio-Palmieri A.C., Borges K.M., Souza J.M.D., Targon M.L. (2007). Terpene production in the peel of sweet orange fruits. Genet. Mol..

[49] Javed S., Javaid A., Mahmood Z., Javaid A., Nasim F. (2011). Biocidal activity of citrus peel essential oils against some food spoilage bacteria. J. Med. Plant Res..

[50] Mahizan N.A., Yang S.K., Moo C.L., Song A.A.L., Chong C.M., Chong C.W., Abushelaibi A., Lim S.H.E., Lai K.S. (2019). Terpene derivatives as a potential agent against antimicrobial resistance (AMR) pathogens. Molecules.

[51] Fisher K., Phillips C. (2008). Potential antimicrobial uses of essential oils in food: Is citrus the answer?. Trends Food Sci. Technol..

[52] Kalemba D.A.A.K., Kunicka A. (2003). Antibacterial and antifungal properties of essential oils. Curr. Med. Chem..

[53] Chanthaphon S., Chanthachum S., Hongpattarakere T. (2008). Antimicrobial activities of essential oils and crude extracts from tropical *Citrus* spp. against food-related microorganisms. Songklanakarin J. Sci. Technol..

[54] Palazzolo E., Laudicina V.A., Germanà M.A. (2013). Current and potential use of citrus essential oils. Curr. Org. Chem..

[55] Dai J., Mumper R.J. (2010). Plant phenolics: Extraction, analysis and their antioxidant and anticancer properties. Molecules.

[56] Papuc C., Goran G.V., Predescu C.N., Nicorescu V., Stefan G. (2017). Plant polyphenols as antioxidant and antibacterial agents for shelf-life extension of meat and meat products: Classification, structures, sources, and action mechanisms. Compr. Rev. Food Sci. Food Saf..

[57] Häkkinen S.H., Kärenlampi S.O., Heinonen I.M., Mykkänen H.M., Törrönen A.R. (1999). Content of the flavonols quercetin, myricetin, and kaempferol in 25 edible berries. J. Agric. Food Chem..

[58] Gu L., Kelm M.A., Hammerstone J.F., Beecher G., Holden J., Haytowitz D., Gebhardt S., Prior R.L. (2004). Concentrations of proanthocyanidins in common foods and estimations of normal consumption. J. Nutr..

[59] Liggins J., Bluck L.J., Runswick S., Atkinson C., Coward W.A., Bingham S.A. (2000). Daidzein and genistein content of fruits and nuts. J. Nutr. Biochem..

[60] Hong H., Landauer M.R., Foriska M.A., Ledney G.D. (2006). Antibacterial activity of the soy isoflavone genistein. J. Basic Microbiol..

[61] Mukne A.P., Viswanathan V., Phadatare A.G. (2011). Structure pre-requisites for isoflavones as effective antibacterial agents. Pharmacogn. Rev..

[62] Selma M.V., Espin J.C., Tomas-Barberan F.A. (2009). Interaction between phenolics and gut microbiota: Role in human health. J. Agric. Food Chem..

[63] Harmand M.F., Blanquet P. (1978). The fate of total Flavanolic Oligomers (OFT) extracted from “*Vitis vinifera* L.” in the rat. Eur. J. Drug Metab. Pharmacokinet..

[64] Howell A.B. (2002). Cranberry proanthocyanidins and the maintenance of urinary tract health. Crit. Rev. Food Sci. Nutr..

[65] Lamy E., Pinheiro C., Rodrigues L., Capela-Silva F., Lopes O., Tavares S., Gaspar R., Combs C.A. (2016). Determinants of tannin-rich food and beverage consumption: Oral perception vs. psychosocial aspects. Tannins: Biochemistry, Food Sources and Nutritional Properties.

[66] Riihinen K., Jaakola L., Kärenlampi S., Hohtola A. (2008). Organ-specific distribution of phenolic compounds in bilberry (*Vaccinium myrtillus*) and ‘northblue’blueberry (*Vaccinium corymbosum* x *V. angustifolium*). Food Chem..

[67] Teixeira J., Gaspar A., Garrido E.M., Garrido J., Borges F. (2013). Hydroxycinnamic acid antioxidants: An electrochemical overview. BioMed Res. Int..

[68] Khoo C., Falk M., Watson R.R., Preedy V.R., Zibadi S. (2014). Polyphenols in the prevention and treatment of vascular and cardiac disease, and cancer. Polyphenols in Human Health and Disease.

[69] Mazur W.M., Uehara M., Wähälä K., Adlercreutz H. (2000). Phyto-oestrogen content of berries, and plasma concentrationsand urinary excretion of enterolactone after asingle strawberry-meal in human subjects. Br. J. Nutr..

[70] Pedraza-Chaverri J., Cárdenas-Rodríguez N., Orozco-Ibarra M., Pérez-Rojas J.M. (2008). Medicinal properties of mangosteen (*Garcinia mangostana*). Food Chem. Toxicol..

[71] Sir Elkhatim K.A., Elagib R.A., Hassan A.B. (2018). Content of phenolic compounds and vitamin C and antioxidant activity in wasted parts of Sudanese citrus fruits. Food Sci. Nutr..

[72] Septembre-Malaterre A., Stanislas G., Douraguia E., Gonthier M.P. (2016). Evaluation of nutritional and antioxidant properties of the tropical fruits banana, litchi, mango, papaya, passion fruit and pineapple cultivated in Réunion French Island. Food Chem..

[73] Määttä K.R., Kamal-Eldin A., Törrönen A.R. (2003). High-performance liquid chromatography (HPLC) analysis of phenolic compounds in berries with diode array and electrospray ionization mass spectrometric (MS) detection: Ribes species. J. Agric. Food Chem..

[74] Tomiyama K., Mukai Y., Saito M., Watanabe K., Kumada H., Nihei T., Hamada N., Teranaka T. (2016). Antibacterial action of a condensed tannin extracted from astringent persimmon as a component of food addictive pancil PS-M on oral polymicrobial biofilms. BioMed Res. Int..

[75] Foo L.Y., Lu Y., Howell A.B., Vorsa N. (2000). A-Type proanthocyanidin trimers from cranberry that inhibit adherence of uropathogenic P-Fimbriated *Escherichia coli*. J. Nat. Prod..

[76] Howell A.B., Reed J.D., Krueger C.G., Winterbottom R., Cunningham D.G., Leahy M. (2005). A-type cranberry proanthocyanidins and uropathogenic bacterial anti-adhesion activity. Phytochemistry.

[77] Rane H.S., Bernardo S.M., Howell A.B., Lee S.A. (2014). Cranberry-derived proanthocyanidins prevent formation of *Candida albicans* biofilms in artificial urine through biofilm-and adherence-specific mechanisms. J. Antimicrob. Chemother..

[78] Vostalova J., Vidlar A., Simanek V., Galandakova A., Kosina P., Vacek J., Vrbkova J., Zimmermann B.F., Ulrichova J., Student V. (2015). Are high proanthocyanidins key to cranberry efficacy in the prevention of recurrent urinary tract infection?. Phytother. Res..

[79] Ribić R., Meštrović T., Neuberg M., Kozina G. (2018). Effective anti-adhesives of uropathogenic *Escherichia coli*. Acta Pharm..

[80] Burdulis D., Sarkinas A., Jasutiene I., Stackevicené E., Nikolajevas L., Janulis V. (2009). Comparative study of anthocyanin composition, antimicrobial and antioxidant activity in bilberry (*Vaccinium myrtillus* L.) and blueberry (*Vaccinium corymbosum* L.) Fruits. Acta Pol. Pharm..

[81] Cisowska A., Wojnicz D., Hendrich A.B. (2011). Anthocyanins as antimicrobial agents of natural plant origin. Nat. Prod. Commun..

[82] Silva S., Costa E.M., Pereira M.F., Costa M.R., Pintado M.E. (2013). Evaluation of the antimicrobial activity of aqueous extracts from dry *Vaccinium corymbosum* extracts upon food microorganism. Food Control.

[83] Lian P.Y., Maseko T., Rhee M., Ng K. (2012). The antimicrobial effects of cranberry against *Staphylococcus aureus*. Food Sci. Technol. Int..

[84] Andrés-Lacueva C., Medina-Remon A., Llorach R., Urpi-Sarda M., Khan N., Chiva-Blanch G., Zamora-Ros R., Rotches-Ribalta M., Lamuela-Raventos R.M., de la Rosa L.A., Alvarez-Parrilla E., González-Aguilar G.A. (2010). Phenolic compounds: Chemistry and occurrence in fruits and vegetables. Fruit and Vegetable Phytochemicals: Chemistry, Nutritional Value and Stability.

[85] Scalbert A. (1991). Antimicrobial properties of tannins. Phytochemistry.

[86] Gaafar A.A., Salama Z.A., Askar M.S., El-Hariri D.M., Bakry B.A. (2013). In Vitro antioxidant and antimicrobial activities of Lignan flax seed extract (*Linum usitatissimum*, L.). Int. J. Pharm. Sci. Rev. Res.

[87] Nohynek L.J., Alakomi H.L., Kähkönen M.P., Heinonen M., Helander I.M., Oksman-Caldentey K.M., Puupponen-Pimiä R.H. (2006). Berry phenolics: Antimicrobial properties and mechanisms of action against severe human pathogens. Nutr. Cancer.

[88] Song X., Liu T., Wang L., Liu L., Li X., Wu X. (2020). Antibacterial effects and mechanism of Mandarin (*Citrus reticulata* L.) essential oil against *Staphylococcus aureus*. Molecules.

[89] Álvarez-Ordóñez A., Carvajal A., Arguello H., Martínez-Lobo F.J., Naharro G., Rubio P. (2013). Antibacterial activity and mode of action of a commercial citrus fruit extract. J. Appl. Microbiol..

[90] Lee K.A., Moon S.H., Kim K.T., Mendonca A.F., Paik H.D. (2010). Antimicrobial effects of various flavonoids on *Escherichia coli* O157: H7 cell growth and lipopolysaccharide production. Food Sci. Biotechnol..

[91] Siriwong S., Thumanu K., Hengpratom T., Eumkeb G. (2015). Synergy and mode of action of Ceftazidime plus Quercetin or Luteolin on *Streptococcus pyogenes*. Evid.-Based Complement. Alternat. Med..

[92] Vikram A., Jayaprakasha G.K., Jesudhasan P.R., Pillai S.D., Patil B.S. (2010). Suppression of bacterial cell–cell signalling, biofilm formation and type III secretion system by citrus flavonoids. J. Appl. Microbiol..

[93] Girennavar B., Cepeda M.L., Soni K.A., Vikram A., Jesudhasan P., Jayaprakasha G.K., Pillai S.D., Patil B.S. (2008). Grapefruit juice and its furocoumarins inhibits autoinducer signaling and biofilm formation in bacteria. Int. J. Food Microbiol..

[94] Vikram A., Jesudhasan P.R., Jayaprakasha G.K., Pillai B.S., Patil B.S. (2010). Grapefruit bioactive limonoids modulate *E. coli* O157:H7 TTSS and biofilm. Int. J. Food Microbiol..

[95] Sheng L., Olsen S.A., Hu J., Yue W., Means W.J., Zhu M.J. (2016). Inhibitory effects of grape seed extract on growth, quorum sensing, and virulence factors of CDC “top-six” non-O157 Shiga toxin producing *E. coli*. Int. J. Food Microbiol..

[96] Tang H., Porras G., Brown M.M., Chassagne F., Lyles J.T., Bacsa J., Bacsa J., Horswill A.R., Quave C.L. (2020). Triterpenoid acids isolated from *Schinus terebinthifolia* fruits reduce *Staphylococcus aureus* virulence and abate dermonecrosis. Sci. Rep..

[97] Konishi K., Adachi H., Ishigaki N., Kanamura Y., Adachi I., Tanaka T., Nishioka I., Nonaka G., Horikoshi I. (1993). Inhibitory effects of tannins on NADH dehydrogenases of various organisms. Biol. Pharm. Bull..

[98] Konishi K., Tanaka T. (1999). Inhibitory effects of tannins on the NADH dehydrogenase activity of bovine heart mitochondrial complex I. Biol. Pharm. Bull..

[99] Chen J.C., Chang Y.S., Wu S.L., Chao D.C., Chang C.S., Li C.C., Ho T.Y., Hsiang C.Y. (2007). Inhibition of *Escherichia coli* heat-labile enterotoxin-induced diarrhea by *Chaenomeles speciosa*. J. Ethnopharmacol..

[100] Reddy S., Taylor M., Zhao M., Cherubin P., Geden S., Ray S., Francis D., Teter K. (2013). Grape extracts inhibit multiple events in the cell biology of cholera intoxication. PLoS ONE.

[101] Morinaga N., Iwamaru Y., Yahiro K., Tagashira M., Moss J., Noda M. (2005). Differential activities of plant polyphenols on the binding and internalization of cholera toxin in vero cells. Int. J. Biol. Chem..

[102] Peabody M.A., Laird M.R., Vlasschaert C., Lo R., Brinkman F.S. (2016). PSORTdb: Expanding the bacteria and archaea protein subcellular localization database to better reflect diversity in cell envelope structures. Nucleic Acids Res..

[103] Fitriyanto N.A., Lewa N., Prasetyo R.A., Kurniawati A., Erwanto Y., Bachruddin Z. (2020). Antibacterial activity of Maja fruit extract against *Escherichia coli* and its potential as urease inhibitor for reducing ammonia emission in poultry excreta. IOP Conf. Ser. Earth Environ. Sci..

[104] Knobloch K., Pauli A., Iberl B., Weigand H., Weis N. (1989). Antibacterial and antifungal properties of essential oil components. J. Essent. Oil Res..

[105] Cox S.D., Gustafson J.E., Mann C.M., Markham J.L., Liew Y.C., Hartland R.P., Bell H.C., Warmington J.R., Wyllie S.G. (1998). Tea tree oil causes K+ leakage and inhibits respiration in *Escherichia coli*. Lett. Appl. Microbiol..

[106] O’Bryan C.A., Pendleton S.J., Crandall P.G., Ricke S.C. (2015). Potential of plant essential oils and their components in animal agriculture–in vitro studies on antibacterial mode of action. Front. Vet. Sci..

[107] Das M.C., Das A., Samaddar S., Daware A.V., Ghosh C., Acharjee S., Sandhu P., Jawed J.J., De U.C., Majumdar S. (2018). Vitexin alters *Staphylococcus aureus* surface hydrophobicity to interfere with biofilm formation. bioRxiv.

[108] Booth I.R. (1985). Regulation of cytoplasmic pH in bacteria. Microbiol. Rev..

[109] Dibner J.J., Buttin P. (2002). Use of organic acids as a model to study the impact of gut microflora on nutrition and metabolism. J. Appl. Poult. Res..

[110] Lu H.J., Breidt F., Pérez-Díaz I.M., Osborne J.A. (2011). Antimicrobial effects of weak acids on the survival of *Escherichia coli* O157:H7 under anaerobic conditions. J. Food Prot..

[111] Salmond C.V., Kroll R.G., Booth I.R. (1984). The effect of food preservatives on pH homeostasis in *Escherichia coli*. Microbiology.

[112] Walter A., Gutknecht J. (1984). Monocarboxylic acid permeation through lipid bilayer membranes. J. Membr. Biol..

[113] Stern J.L., Hagerman A.E., Steinberg P.D., Mason P.K. (1996). Phlorotannin-protein interactions. J. Chem. Ecol..

[114] Shen X., Sun X., Xie Q., Liu H., Zhao Y., Pan Y., Hwang C.A., Wu V.C. (2014). Antimicrobial effect of blueberry (*Vaccinium corymbosum* L.) extracts against the growth of *Listeria monocytogenes* and *Salmonella* Enteritidis. Food Control..

[115] Flemming H.C., Wingender J. (2010). The biofilm matrix. Nat. Rev. Microbiol..

[116] Amrutha B., Sundar K., Shetty P.H. (2017). Effect of organic acids on biofilm formation and quorum signaling of pathogens from fresh fruits and vegetables. Microb. Pathog..

[117] Almasoud A., Hettiarachchy N., Rayaprolu S., Babu D., Kwon Y.M., Mauromoustakos A. (2016). Inhibitory effects of lactic and malic organic acids on autoinducer type 2 (AI-2) quorum sensing of *Escherichia coli* O157:H7 and *Salmonella typhimurium*. LWT Food Sci. Technol..

[118] Singla R., Goel H., Ganguli A. (2014). Novel synergistic approach to exploit the bactericidal efficacy of commercial disinfectants on the biofilms of *Salmonella enterica* serovar Typhimurium. J. Biosci. Bioeng..

[119] Espina L., Somolinos M., Lorán S., Conchello P., García D., Pagán R. (2011). Chemical composition of commercial citrus fruit essential oils and evaluation of their antimicrobial activity acting alone or in combined processes. Food Control.

[120] Laird K., Armitage D., Phillips C. (2012). Reduction of surface contamination and biofilms of *Enterococcus* sp. and *Staphylococcus aureus* using a citrus-based vapour. J. Hosp. Infect..

[121] Espina L., Pagán R., López D., García-Gonzalo D. (2015). Individual constituents from essential oils inhibit biofilm mass production by multi-drug resistant *Staphylococcus aureus*. Molecules.

[122] Chen Y., Liu T., Wang K., Hou C., Cai S., Huang Y., Du Z., Huang H., Kong J., Chen Y. (2016). Baicalein inhibits *Staphylococcus aureus* biofilm formation and the quorum sensing system in vitro. PLoS ONE.

[123] Bakkiyaraj D., Nandhini J.R., Malathy B., Pandian S.K. (2013). The anti-biofilm potential of pomegranate (*Punica granatum* L.) extract against human bacterial and fungal pathogens. Biofouling.

[124] Zhong Z., Yu X., Zhu J. (2008). Red bayberry extract inhibits growth and virulence gene expression of the human pathogen *Vibrio cholerae*. J. Antimicrob. Chemother..

[125] Zhu J., Miller M.B., Vance R.E., Dziejman M., Bassler B.L., Mekalanos J.J. (2002). Quorum-sensing regulators control virulence gene expression in *Vibrio cholerae*. Proc. Natl. Acad. Sci. USA.

[126] Opoku-Temeng C., Sintim H.O. (2016). Inhibition of cyclic diadenylate cyclase, DisA, by polyphenols. Sci. Rep..

[127] Schuier M., Sies H., Illek B., Fischer H. (2005). Cocoa-related flavonoids inhibit CFTR-mediated chloride transport across T84 human colon epithelia. J. Nutr..

[128] Lescano C.H., de Oliveira I.P., Zaminelli T., Baldivia D.D.S., da Silva L.R., Napolitano M., Silvério C.B.M., Lincopan N., Sanjinez-Argandona E.J. (2016). *Campomanesia adamantium* peel extract in antidiarrheal activity: The ability of inhibition of heat-stable enterotoxin by polyphenols. PLoS ONE.

[129] Pellarin M.G., Albrecht C., Rojas M.J., Aguilar J.J., Konigheim B.S., Paraje M.G., Albesa I., Eraso A.J. (2013). Inhibition of cytotoxicity of Shiga toxin of *Escherichia coli* O157: H7 on vero cells by *Prosopis alba* Griseb (Fabaceae) and *Ziziphus mistol* Griseb (Rhamnaceae) extracts. J. Food Prot..

[130] Cherubin P., Garcia M.C., Curtis D., Britt C.B., Craft J.W., Burress H., Berndt C., Reddy S., Guyette J., Zheng T. (2016). Inhibition of cholera toxin and other AB toxins by polyphenolic compounds. PLoS ONE.

[131] World Health Organization (WHO) Antimicrobial Resistance. https://www.who.int/news-room/fact-sheets/detail/antimicrobial-resistance.

[132] Cheesman M.J., White A., Matthews B., Cock I.E. (2019). *Terminalia ferdinandiana* fruit and leaf extracts inhibit methicillin-resistant *Staphylococcus aureus* growth. Planta Med..

[133] Dharmaratne M.P.J., Manoraj A., Thevanesam V., Ekanayake A., Kumar N.S., Liyanapathirana V., Abeyratne E., Bandara B.R. (2018). *Terminalia bellirica* fruit extracts: In-vitro antibacterial activity against selected multidrug-resistant bacteria, radical scavenging activity and cytotoxicity study on BHK-21 cells. BMC Complement. Altern. Med..

[134] Chaves-López C., Usai D., Donadu M.G., Serio A., González-Mina R.T., Simeoni M.C., Molicotti P., Zanetti S., Pinna A., Paparella A. (2018). Potential of *Borojoa patinoi* Cuatrecasas water extract to inhibit nosocomial antibiotic resistant bacteria and cancer cell proliferation in vitro. Food Funct..

[135] Viktorová J., Kumar R., Řehořová K., Hoang L., Ruml T., Figueroa C.R., Valdenegro M., Fuentes L. (2020). Antimicrobial activity of extracts of two native fruits of Chile: Arrayan (*Luma apiculata*) and Peumo (*Cryptocarya alba*). Antibiotics.

[136] Dey D., Ray R., Hazra B. (2015). Antimicrobial activity of pomegranate fruit constituents against drug-resistant *Mycobacterium tuberculosis* and β-lactamase producing *Klebsiella pneumoniae*. Pharm. Biol..

[137] Li Z., Summanen P.H., Komoriya T., Henning S.M., Lee R.P., Carlson E., Heber D., Finegold S.M. (2015). Pomegranate ellagitannins stimulate growth of gut bacteria in vitro: Implications for prebiotic and metabolic effects. Anaerobe.

[138] Chen M.L., Yi L., Zhang Y., Zhou X., Ran L., Yang J., Zhu J.D., Zhang Q.Y., Mi M.T. (2016). Resveratrol attenuates trimethylamine-N-oxide (TMAO)-induced atherosclerosis by regulating TMAO synthesis and bile acid metabolism via remodeling of the gut microbiota. mBio.

[139] Hidalgo M., Oruna-Concha M.J., Kolida S., Walton G.E., Kallithraka S., Spencer J.P., de Pascual-Teresa S. (2012). Metabolism of anthocyanins by human gut microflora and their influence on gut bacterial growth. J. Agric. Food Chem..

[140] Riaz Rajoka M.S., Thirumdas R., Mehwish H.M., Umair M., Khurshid M., Hayat H.F., Phimolsiripol Y., Pallarés N., Martí-Quijal F.J., Barba F.J. (2021). Role of food antioxidants in modulating gut microbial communities: Novel understandings in intestinal oxidative stress damage and their impact on host health. Antioxidants.

[141] Deledda A., Annunziata G., Tenore G.C., Palmas V., Manzin A., Velluzzi F. (2021). Diet-derived antioxidants and their role in inflammation, obesity and gut microbiota modulation. Antioxidants.

[142] Plamada D., Vodnar D.C. (2021). Polyphenols—Gut microbiota interrelationship: A transition to a new generation of prebiotics. Nutrients.

[143] Shahidi F., Janitha P.K., Wanasundara P.D. (1992). Phenolic antioxidants. Crit. Rev. Food Sci. Nutr..

[144] Saura-Calixto F., Serrano J., Pérez-Jiménez J., Preedy V.R. (2009). What contribution is beer to the intake of antioxidants in the diet?. Beer in Health and Disease Prevention.

[145] Rojas J., Buitrago A., Segura-Campos M.R. (2019). Antioxidant activity of phenolic compounds biosynthesized by plants and its relationship with prevention of neurodegenerative diseases. Bioactive Compounds.

[146] Rubio C.P., Hernández-Ruiz J., Martinez-Subiela S., Tvarijonaviciute A., Ceron J.J. (2016). Spectrophotometric assays for total antioxidant capacity (TAC) in dog serum: An update. BMC Vet. Res..

[147] Denardin C.C., Hirsch G.E., da Rocha R.F., Vizzotto M., Henriques A.T., Moreira J.C., Guma F.T., Emanuelli T. (2015). Antioxidant capacity and bioactive compounds of four Brazilian native fruits. J. Food Drug Anal..

[148] Ribeiro L.D.O., de Freitas B.P., Lorentino C.M.A., Frota H.F., Dos Santos A.L.S., Moreira D.D.L., Amaral B.S.D., Jung E.P., Kunigami C.N. (2022). Umbu fruit peel as source of antioxidant, antimicrobial and α-amylase inhibitor compounds. Molecules.

[149] Shan S., Huang X., Shah M.H., Abbasi A.M. (2019). Evaluation of polyphenolics content and antioxidant activity in edible wild fruits. BioMed Res. Int..

[150] Batiston W.P., Maruyama S.A., Gomes S.T.M., Visentainer J.V., de Souza N.E., Matsushita M. (2013). Total phenolic content and antioxidant capacity of methanolic extracts of ten fruits. Acta Sci. Technol..

[151] Hidalgo G.I., Almajano M.P. (2017). Red fruits: Extraction of antioxidants, phenolic content, and radical scavenging determination: A review. Antioxidants.

[152] Huang W.Y., Zhang H.C., Liu W.X., Li C.Y. (2012). Survey of antioxidant capacity and phenolic composition of blueberry, blackberry, and strawberry in Nanjing. J. Zhejiang Univ. Sci. B.

[153] Bernal-Gallardo J.O., Molina-Torres J., Angoa-Pérez M.V., Cárdenas-Valdovinos J.G., García-Ruíz I., Ceja-Díaz J.A., Mena-Violante H.G. (2021). Phenolic compound content and the antioxidant and antimicrobial activity of wild blueberries (*Vaccinium stenophyllum* steud.) fruits extracts during ripening. Horticulturae.

[154] Olas B. (2018). Berry phenolic antioxidants–implications for human health?. Front. Pharmacol..

[155] Zorzi M., Gai F., Medana C., Aigotti R., Morello S., Peiretti P.G. (2020). Bioactive compounds and antioxidant capacity of small berries. Foods.

[156] Lobo V., Patil A., Phatak A., Chandra N. (2010). Free radicals, antioxidants and functional foods: Impact on human health. Pharmacogn. Rev..

[157] Najafabad A.M., Jamei R. (2014). Free radical scavenging capacity and antioxidant activity of methanolic and ethanolic extracts of plum (*Prunus domestica* L.) in both fresh and dried samples. Avicenna J. Phytomed..

[158] Mathew S., Abraham T.E., Zakaria Z.A. (2015). Reactivity of phenolic compounds towards free radicals under in vitro conditions. J. Food Sci. Technol..

[159] Siquet C., Paiva-Martins F., Lima J.L., Reis S., Borges F. (2006). Antioxidant profile of dihydroxy-and trihydroxyphenolic acids—A structure–activity relationship study. Free Radic. Res..

[160] Chen J.W., Zhu Z.Q., Hu T.X., Zhu D.Y. (2002). Structure-activity relationship of natural flavonoids in hydroxyl radical-scavenging effects. Acta Pharmacol. Sin..

[161] Sukmaningsih A.A.S.A., Permana S., Santjojo D.J.D.H., Wardoyo A.Y.P., Sumitro S.B. (2018). Investigating natural transition metal coordination anthocyanin complex in java plum (*Syzygium cumini*) fruit as free radical scavenging. Rasayan J. Chem..

[162] Malešev D., Kuntić V. (2007). Investigation of metal-flavonoid chelates and the determination of flavonoids via metal-flavonoid complexing reactions. J. Serbian Chem. Soc..

[163] De Souza R.F., De Giovani W.F. (2004). Antioxidant properties of complexes of flavonoids with metal ions. Redox Rep..

[164] Baysal T., Demirdöven A. (2007). Lipoxygenase in fruits and vegetables: A review. Enzyme Microb. Technol..

[165] Veldink G.A., Hilbers M.P., Nieuwenhuizen W.F., Vliegenthart J.F.G. (1999). Plant lipoxygenase: Structure and mechanism. Eicosanoids and Related Compounds in Plants and Animals.

[166] Salas J.J., Sánchez C., García-González D.L., Aparicio R. (2005). Impact of the suppression of lipoxygenase and hydroperoxide lyase on the quality of the green odor in green leaves. J. Agric. Food Chem..

[167] Alam F., us Saqib Q.N., Ashraf M. (2017). *Gaultheria trichophylla* (Royle): A source of minerals and biologically active molecules, its antioxidant and anti-lipoxygenase activities. BMC Complement Altern. Med..

[168] Berkoz M. (2020). Antioxidant and anti-lipoxygenase activities of *Cydonia oblonga*. Medicine.

[169] Kohyama N., Nagata T., Fujimoto S.I., Sekiya K. (1997). Inhibition of arachidonate lipoxygenase activities by 2-(3, 4-dihydroxyphenyl) ethanol, a phenolic compound from olives. Biosci. Biotechnol. Biochem..

[170] Szymanowska U., Baraniak B., Bogucka-Kocka A. (2018). Antioxidant, anti-inflammatory, and postulated cytotoxic activity of phenolic and anthocyanin-rich fractions from polana raspberry (*Rubus idaeus* L.) fruit and juice—In vitro study. Molecules.

[171] Purkiewicz A., Pietrzak-Fiećko R. (2021). Antioxidant properties of fruit and vegetable whey beverages and fruit and vegetable mousses. Molecules.

[172] Vundela S.R., Kalagatur N.K., Nagaraj A., Krishna K., Chandranayak S., Kondapalli K., Hashem A., Abd_Allah E.F., Poda S. (2021). Multi-biofunctional properties of phytofabricated selenium nanoparticles from *Carica papaya* fruit extract: Antioxidant, antimicrobial, antimycotoxin, anticancer, and biocompatibility. Front. Microbiol..

[173] Abdel-Naeem H.H., Elshebrawy H.A., Imre K., Morar A., Herman V., Pașcalău R., Sallam K.I. (2022). Antioxidant and antibacterial effect of fruit peel powders in chicken patties. Foods.

[174] Etienne-Mesmin L., Chassaing B., Desvaux M., De Paepe K., Gresse R., Sauvaitre T., Forano E., Van de Wiele T., Schüller S., Juge N. (2019). Experimental models to study intestinal microbes–mucus interactions in health and disease. FEMS Microbiol. Rev..

[175] Fournier E., Roussel C., Dominicis A., Ley D., Peyron M.A., Collado V., Mercier-Bonin M., Lacroix C., Alric M., Van de Wiele T. (2021). In vitro models of gut digestion across childhood: Current developments, challenges and future trends. Biotechnol. Adv..

